# A survey of optimal strategy for signature-based drug repositioning and an application to liver cancer

**DOI:** 10.7554/eLife.71880

**Published:** 2022-02-22

**Authors:** Chen Yang, Hailin Zhang, Mengnuo Chen, Siying Wang, Ruolan Qian, Linmeng Zhang, Xiaowen Huang, Jun Wang, Zhicheng Liu, Wenxin Qin, Cun Wang, Hualian Hang, Hui Wang

**Affiliations:** 1 State Key Laboratory of Oncogenes and Related Genes, Department of Liver Surgery & Shanghai Cancer Institute, Renji Hospital, Shanghai Jiao Tong University School of Medicine Shanghai China; 2 Key Laboratory of Gastroenterology and Hepatology, Division of Gastroenterology and Hepatology, Renji Hospital, Shanghai Jiao Tong University School of Medicine Shanghai China; 3 Hepatic Surgery Center, Tongji Hospital, Tongji Medical College, Huazhong University of Science and Technology Wuhan China; Flinders Medical Centre Australia; McGill University Canada

**Keywords:** drug prediction, connectivity map, LINCS, liver cancer, homoharringtonine, Human

## Abstract

Pharmacologic perturbation projects, such as Connectivity Map (CMap) and Library of Integrated Network-based Cellular Signatures (LINCS), have produced many perturbed expression data, providing enormous opportunities for computational therapeutic discovery. However, there is no consensus on which methodologies and parameters are the most optimal to conduct such analysis. Aiming to fill this gap, new benchmarking standards were developed to quantitatively evaluate drug retrieval performance. Investigations of potential factors influencing drug retrieval were conducted based on these standards. As a result, we determined an optimal approach for LINCS data-based therapeutic discovery. With this approach, homoharringtonine (HHT) was identified to be a candidate agent with potential therapeutic and preventive effects on liver cancer. The antitumor and antifibrotic activity of HHT was validated experimentally using subcutaneous xenograft tumor model and carbon tetrachloride (CCL_4_)-induced liver fibrosis model, demonstrating the reliability of the prediction results. In summary, our findings will not only impact the future applications of LINCS data but also offer new opportunities for therapeutic intervention of liver cancer.

## Introduction

Despite the major advances in drug research and development (R&D), the cost for de novo drug development remains high, ranging from $3 billion to more than $30 billion. Moreover, it usually takes over 10 years to bring a new drug from bench to bedside, reflecting the complex challenges in this area (Scannell et al., 2012). Within this context, exploring new indications for existing drugs (drug-centric) or identifying effective drugs for certain diseases (disease-centric) represents an appealing concept, namely ‘drug repositioning’ (or ‘drug repurposing’), which can greatly shorten the gap between preclinical drug research and clinical applications (Ashburn and Thor, 2004; Liu et al., 2013). Leveraging big data-driven approaches, drug repositioning can be conducted computationally, which has the potential to complement traditional therapeutic discovery means and further improve the cost-effectiveness of drug development (Li et al., 2016). The most notable data resources supporting the in silico-based therapeutic discovery campaigns would be the Connectivity Map (CMap) (Lamb et al., 2006) and its recent extension called Library of Integrated Network-Based Cellular Signatures (LINCS) (Subramanian et al., 2017). These two projects have generated large-scale drug-induced gene expression profiles on multiple cancer cell lines under different treatment conditions (CMap Build 2: 3 cell lines, 1309 compounds; LINCS: 77 cell lines, 19,811 compounds), representing a treasure trove for in silico therapeutic exploration (Musa et al., 2018). As a 1000-fold scale-up of the original CMap, LINCS contained dramatic increases in both cell line types and perturbations, making it the focus of the present investigation.

**Figure 1.**
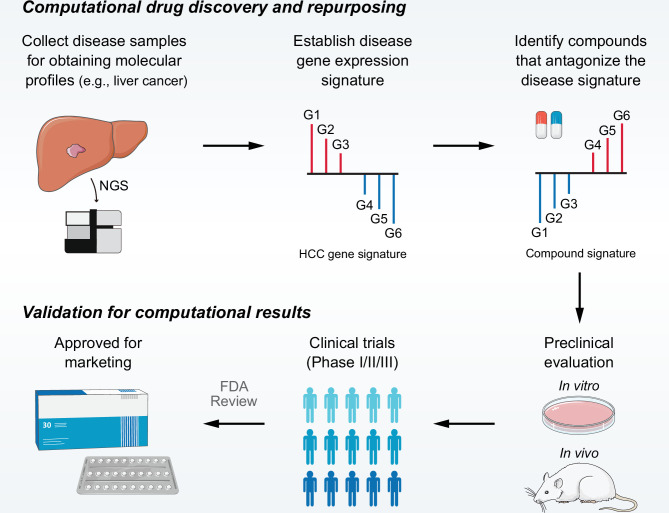
Overview of LINCS data-driven therapeutic discovery. The working principle of ‘signature reversion’-based computational approach. A disease signature representing discordant expression pattern needs first to be identified (G1, G2, and G3 stand for upregulated genes while G4, G5, and G6 stand for down-regulated genes in disease state). With this signature, pharmacologic perturbation data sets can be queried to find compounds with the ability to reverse disease expression pattern (suppress expression of G1, G2, and G3 and induce expression of G4, G5, and G6). After determining the candidate compounds, experimental and clinical validation are required to translate computational findings to clinical applications. LINCS, Library of Integrated Network-based Cellular Signatures.

The computational drug discovery approach using LINCS (also CMap) data is based upon a basic concept called ‘signature reversion’ (Li et al., 2016). Briefly, compounds with the ability to reverse disease-specific gene expression pattern are considered therapeutic candidates (Figure 1). To date, although there have been many successful applications, many problems with this approach remain unsolved (Chen et al., 2017a; Chen et al., 2017b; van Noort et al., 2014). Due to the lack of appropriate benchmarking standards, limited studies have investigated the factors influencing the accuracy of this approach. Therefore, no consensus regarding the implementation details has been reached across current studies. Constructing rational benchmarking standards and developing the best practice approach can facilitate the development of signature reversion approach and help to identify more effective therapeutic strategies for refractory diseases.

**Figure 1—figure supplement 1.**
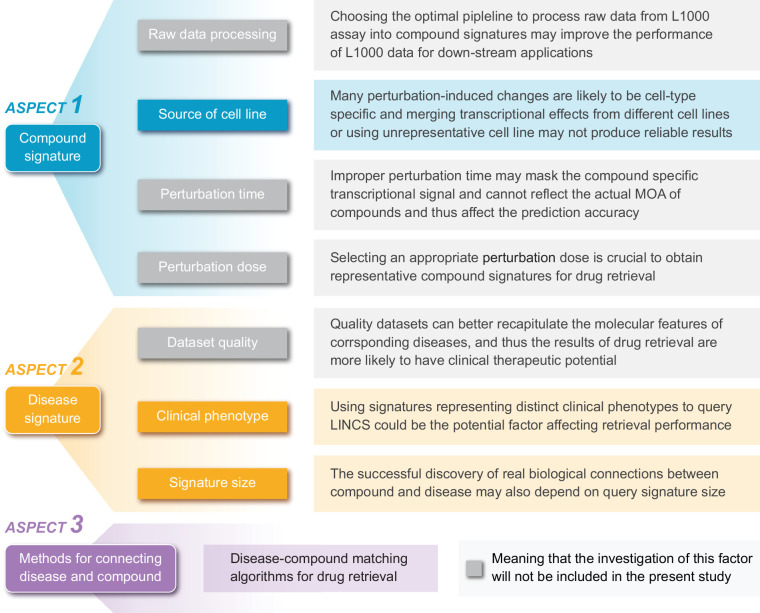
A summary of potential factors influencing the accuracy of signature reversion-based computational approach. Within the framework of this approach, there are mainly three components: compound signature, disease signature, and signature matching methods. Each component is likely to be influenced by several factors. In addition to the brief descriptions illustrated in this figure, we also discussed these factors in more detail in Supplementary Discussion.

**Figure 1—figure supplement 2.**
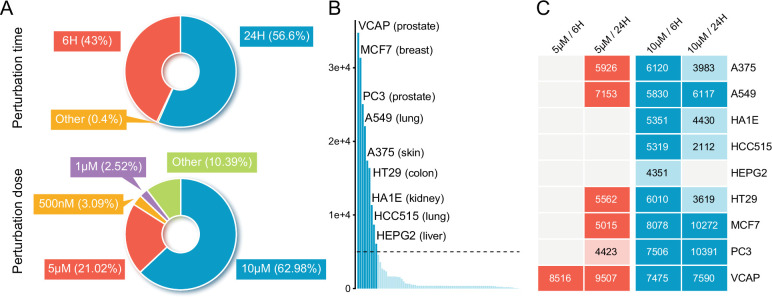
An overview of compound-induced expression profiles in LINCS. (**A**) The distribution of compound profiles of different perturbation times (upper) and concentrations (lower) across all the compound experiments in LINCS data set. (**B**) The profile count distribution of all 71 cell lines in LINCS. Each bar represents the number of available compound profiles per cell line. The nine most profiled cell lines were labeled in the figure. (**C**) Heatmap integrating annotation of the cell lines with perturbation time and concentration. The specific values have not been displayed if there are less than 2000 profiles in the combination of cell line and experimental conditions. LINCS, Library of Integrated Network-based Cellular Signatures.

Herein, we mainly focused on the disease of liver cancer. As one of the most lethal malignancies worldwide, liver cancer directly accounts for nearly one million deaths each year (Bray et al., 2018). Hepatocellular carcinoma (HCC) is the major type of liver cancer, representing approximately 90% of all liver cancer cases (Llovet et al., 2016). Although many standard of care therapies, including Lenvatinib (Kudo et al., 2018), regorafenib (Bruix et al., 2017), cabozantinib (Abou-Alfa et al., 2018), ramucirumab (Zhu et al., 2019), pembrolizumab (Finn et al., 2020b), nivolumab (El-Khoueiry et al., 2017), and atezolizumab-bevacizumab (Finn et al., 2020a), have been approved for treating HCC in recent years, most of them can yield only marginal survival benefit. Thus, more effective therapeutics treatments for HCC are highly desired. The objectives of the present study were threefold. The first objective was to develop novel benchmarking standards for evaluating drug retrieval performance. The second one was to determine the best practice approach for LINCS data-based signature reversion. For the last objective, we sought to identify novel drug candidates against liver cancer, exploiting the findings from the second objective.

## Results

### Summary of influencing factors and compound experiments in LINCS

Many factors may affect the accuracy of signature-based drug retrieval. We have categorized these factors into three main aspects: acquisition of compound signature (reference signature), generation of disease signature (query signature), and selection of disease-compound matching methods (Figure 1, Figure 1—figure supplement 1). Although all factors were mentioned and discussed, not all of them were included in the present analyses, considering that some factors have been covered elsewhere and some were challenging to explore due to data and method restrictions. In this study, systematic analyses were carried out to assess the influences of four major factors on signature matching-based drug discovery, including source of cell line, clinical phenotype of query signature, query signature size, and signature matching method.

Since only compound-induced expression data was the focus of this study, we first excluded experiments of other perturbagens, including gene knockdown (or knockout) and gene overexpression manipulations. Subsequently, the distribution of compound profiles was visualized based on their perturbation time, perturbation dose, and cell line used. Most of the measurements were made in the treatment durations of 6 hr (43%) or 24 hr (56.6%), and under the concentrations of 5 μM (21%) or 10 μM (63%) (Figure 1—figure supplement 2A). The count distribution of all cell lines in LINCS was also presented in Figure 1—figure supplement 2B. Although 71 cell lines were included in LINCS project in total, not all of them were extensively profiled, and only 9 cell lines contained more than 5000 profiles, which, however, account for 77.8% of all compound profiles. There were 2912 compounds shared by these nine cell lines. We further integrated annotation of the most profiled cell lines with treatment duration and concentration information, and illustrated the specific profile numbers of each cell line under the conditions of certain time and dose (Figure 1—figure supplement 2C). Unless otherwise indicated, all the following analyses were performed on a fixed perturbation condition of 10 μM for 6 hr. Besides, compound profiles of all top nine cell lines were only utilized when investigating the factor ‘Source of cell line.’ In other cases, we focused exclusively on the cell line of HepG2, as our main point was to uncover novel therapeutics for liver cancer in this study. A systematic summary of included data sets for analyses was presented in Supplementary file 1A and B.

### Compound-induced expression changes are highly cell line-specific

**Figure 2.**
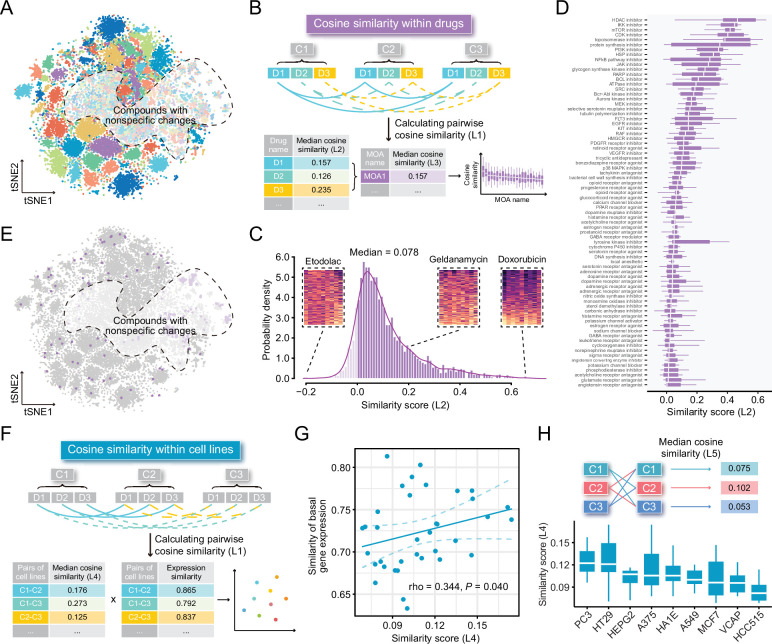
Highly cell-type specific compound-induced expression changes. (**A**) Two-dimensional t-SNE projection based on cosine distance between compound signatures. Each dot represents a unique perturbation-induced expression profile, and each color represents one type of cell line. Drug perturbation data was obtained from GSE92742 and GSE70138. (**B**) Schematic diagram displaying the calculation process of compound-level (L2) and MOA-level (L3) similarity scores. (**C**) Distribution of compound-level (L2) cosine similarity scores, which range from –1 (completely opposite pattern) to 1 (perfectly identical pattern). Three examples are presented (left to right: etodolac, geldanamycin, and doxorubicin). (**D**) Illustration of MOA-level (L3) similarities. Only MOAs with more than five compounds included are shown in the figure. (**E**) A t-SNE projection showing the distribution of compounds (indicated by purple dots) in top ranked five MOAs (including HDAC inhibitors, IKK inhibitors, mTOR inhibitors, CDK inhibitors, and topoisomerase inhibitors). (**F**) Schematic diagram displaying the calculation process of cell line pair-level (L4) similarity scores. (**G**) Correlation between basal expression similarities and perturbed expression similarities (L4) of 36 cell line pairs (nine cell lines in total). Statistical significance and correlation coefficient were determined by ranked-based Spearman correlation. (**H**) Schematic view of the calculation of cell line-level (L5) similarity scores (upper) and the presentation of L5 similarity scores of nine cell lines in the boxplot (lower). Data are presented as median±quartiles. MOA, mechanism of action; t-SNE, t-distributed stochastic neighbor embedding.

Some previous studies utilized compound profiles from cell lines irrelevant to the disease they are studied for signature reversion prediction. To investigate whether this was a reasonable practice, we conducted following analyses based on LINCS data of the nine most profiled cell lines. First, we visualized the compound profiles in a cosine distance-based two-dimensional t-distributed stochastic neighbor embedding (t-SNE) plot that represented the overall compound perturbation space wherein each dot was equivalent to a unique perturbation and each cell line was color-coded (Figure 2A). As shown in the figure, most dots with the same color clustered together, indicating that most of compound-induced gene expression changes tended to be cell-type specific. Intriguingly, dots with different colors in the white region seemed to mix together, suggesting that some compounds might induce similar gene expression changes across cell lines. To figure out which compounds were likely to cause cell-type specific gene expression changes and which tended to induce universal changes independent of cell lines, we calculated the pairwise cosine similarities (L1) among the profiles from the same compounds measured in different cell lines (Figure 2B). The cosine similarity measures range from –1 to 1, where higher values indicate increased similarity. The similarity scores (compound-level, L2) of the 2912 unique compounds were determined by calculating the median pairwise cosine similarity values (L1) across the nine cell lines (Supplementary file 2). As a result, a high degree of cell-specificity was observed for most compounds, with a median L2 similarity score of 0.078 (Figure 2C). Furthermore, we retrieved the mechanism of action (MOA) information and mapped them to the compounds to determine the MOA-level similarity scores (L3). L3 similarity scores were calculated based on the median values of L2 similarity scores of compounds within the same MOA. Results suggested that inhibitors targeting core cellular processes (e.g., cell cycle, RNA transcription, and protein synthesis) tended to induce similar changes across all cell lines, generally in agreement with previous findings (Figure 2D, Supplementary file 2; Niepel et al., 2017; Subramanian et al., 2017; Wang et al., 2018). We then marked the dots representing the compounds of the top five MOAs in the t-SNE plot. As expected, most of marked dots fell in clusters within the nonspecific region (Figure 2E).

**Figure 2—figure supplement 1.**
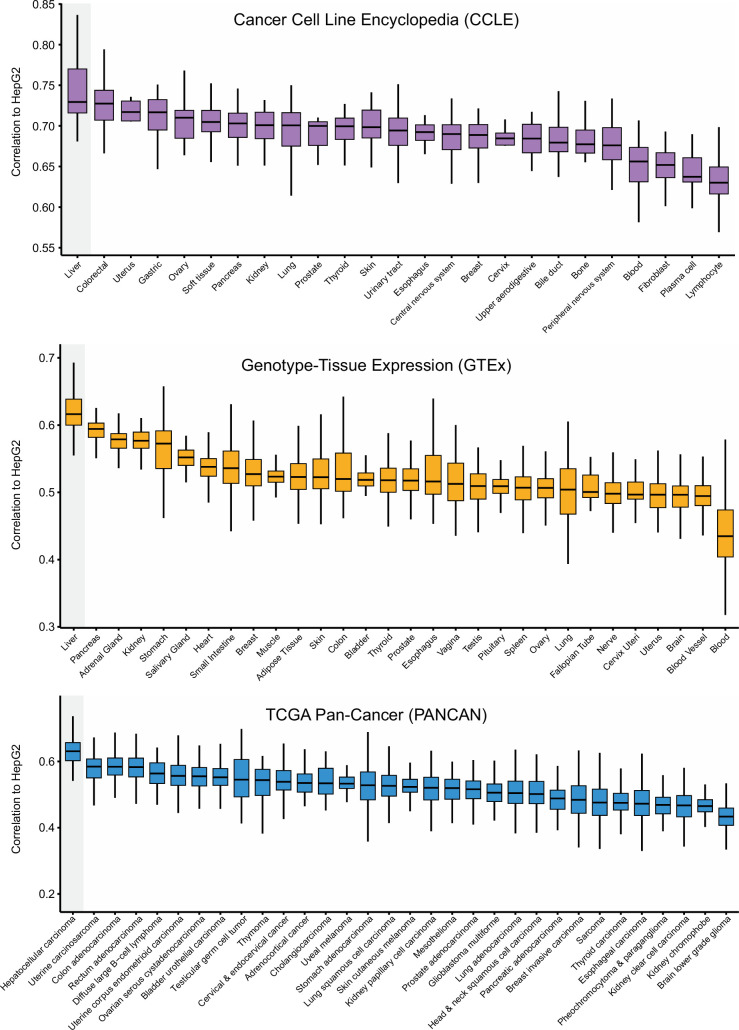
Correlations between HepG2 cell line and other cancer cell lines or normal/tumor tissues. Expression data were derived from CCLE, GTEx, and TCGA Pan-Cancer, respectively. Correlations were determined by ranked-based Spearman correlation analysis. The line within the boxes represents the median value, the bottom and top of the boxes denote the interquartile range, and the vertical line represents 1.5 times the interquartile range.

Apart from investigating the similarity of perturbed expression profiles at compound level, we further sought to further investigate the cell line pair/cell line-level similarity. Nine cell lines contributed a total of 36 unique cell line pairs. The cell line pair-level perturbed expression similarities (L4) were determined through calculating the median value of similarity scores of all compound pairs between two cell lines, and the corresponding basal expression similarities were computed using Spearman ranked correlation on expression data from CCLE project (Figure 2F). The result showed that there was a significant, albeit not very remarkable, association between the perturbed expression similarities (cell line pair-level, L4) and basal expression similarities (ρ=0.344; p=0.040), suggesting that cell lines with similar molecular features were more likely to have consistent gene expression changes upon perturbation (Figure 2G). Similarities within the nine cell lines were also explored (cell line-level, L5). Among the nine cell lines we tested, PC3 cell line showed the highest L5 similarity score (median value=0.122) (Figure 2H). Notably, the cosine similarity of 0.122 still denoted a weak relationship, which further supported the conclusion that compound-induced gene expression changes were highly cell line-specific.

Among the nine most profiled cell lines, HepG2 was the only one derived from liver. To investigate whether HepG2 was an appropriate cell line model for computational therapeutics discovery for liver cancer or other liver-associated diseases, we calculated the expression correlation between HepG2 and other cell lines (921 CCLE cell lines) or tissues (17,382 normal tissues from GTEx and 9701 tumor tissues from TCGA PanCancer). Compared to other tissue-derived cancer cell lines or normal/tumor tissues, HepG2 exhibited a significantly higher expression correlation with liver cancer cell lines (median correlation coefficient=0.729), normal liver tissues (median correlation coefficient=0.616), and liver cancer tissues (median correlation coefficient=0.631) (Figure 2—figure supplement 1). Collectively, we supposed that the use of LINCS-derived HepG2 data was preferable to be limited within liver diseases.

### Developing benchmarking standards for evaluating drug retrieval performance

**Figure 3.**
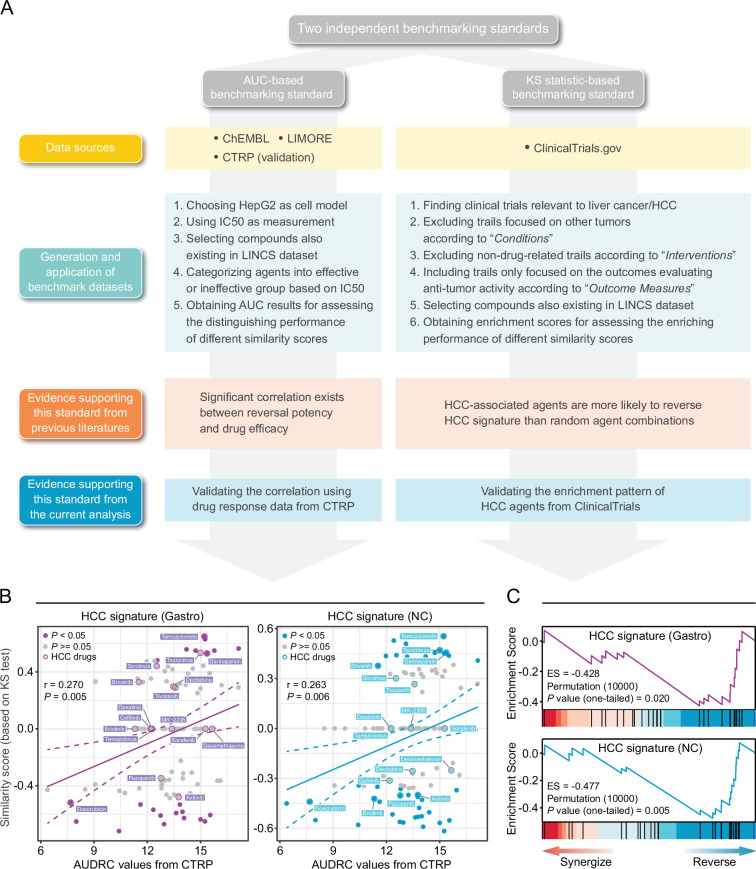
Establishment of novel benchmarking standards. (**A**) Flow chart of the data collection and hypothesis validation for the AUC-based (left) and KS statistic-based (right) benchmarking standards. (**B**) Correlation between drug efficacy (AUDRC values) and reversal potency (KS-based similarity scores). Two previously published query signatures, including Sig_gastro_ (left) and Sig_NC_ (right), were utilized to calculate similarity scores. Drug response data was achieved from CTRP data set. Note that lower similarity scores indicate higher reversal potency and lower AUDRC values imply greater drug sensitivity. Color toward gray indicates no statistical significance determined by KS test. (**C**) Reversal potency of HCC agents demonstrated by enrichment analysis. Sig_gastro_ (upper) and Sig_NC_ (lower) were used to compute similarity scores. AUC, area under the curve; HCC, hepatocellular carcinoma; KS, Kolmogorov-Smirnov.

Owing to the lack of benchmarking standards, accurate assessment of retrieval performance of signature matching methods remains challenging. Inspired by previous findings (Chen et al., 2017a; Chen et al., 2017b; Cheng et al., 2014; Wagner et al., 2015), we proposed two novel benchmarking standards, namely area under the curve (AUC)-based standard and Kolmogorov-Smirnov (KS) statistic-based standard. They were built upon different notions and thus were independent of each other, which helped to avoid potential bias introduced by single standard. The corresponding benchmarking data sets were developed mainly based on preclinical/clinical data of liver cancer. Detailed processes of data collection and metrics calculation were described in Materials and methods and visualized in Figure 3A.

The development of AUC-based standard was based on the finding that there existed correlation between the reversal potency and treatment efficacy (Chen et al., 2017a; Wagner et al., 2015). In order to further validate whether this correlation remained significant in other conditions, we retrieved drug response data from CTRP data set in which area under the dose-response curve (AUDRC) values were used as measurements of drug sensitivity, and utilized two different HCC signatures as query signatures to obtain KS-based similarity scores (Chen et al., 2017a; Chen et al., 2017b). A total of 109 compounds shared by two data sets were selected to conduct correlation analyses. As a result, statistically significant correlation could still be observed between similarity scores and AUDRC values in these scenarios, further proving the reliability of this standard (Figure 3B). A benchmark data set was then generated, composed of 117 unique compounds with both LINCS and drug efficacy (IC_50_) data available, which was taken as a basis for the application of AUC-based standard (Supplementary file 3). The resultant AUC from this standard was termed as drug retrieval-associated AUC (DR-AUC). Higher DR-AUC value indicated better performance.

As for KS statistic-based standard, we assumed that agents under evaluation in clinical trial for HCC treatment, namely HCC-related agents, might possess an increased reversal capacity (Chen et al., 2017b). In other words, HCC agents were more likely to cause negative enrichment in KS test. To verify this hypothesis, we compiled a set of 27 potential HCC agents which were both included in LINCS and under clinical trials for liver cancer treatment. Besides, similarity scores of all compounds tested in HepG2 were also calculated, which were then used as ranked list for KS test. The results of KS test demonstrated that the HCC agent set was indeed negatively enriched (Figure 3C). The resultant enrichment scores (ES) here were termed as drug retrieval-associated ES (DR-ES) (Supplementary file 4). Of note, in contrast to DR-AUC, lower DR-ES values denoted better performance.

### XSum is the optimal signature matching method for drug retrieval

**Figure 4.**
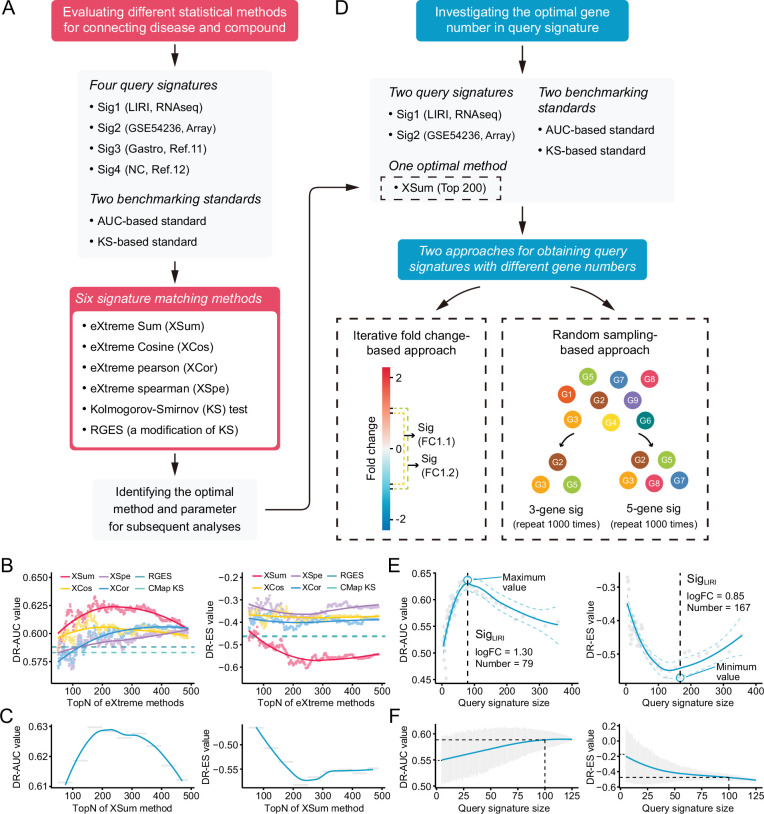
Benchmarking different methodologies and parameters. (**A**) Diagram summarizing the workflow and the important components involved in the evaluation process of drug retrieval performance of six different signature matching methods. (**B**) Retrieval performance of six matching methods evaluated by AUC-based benchmarking standard (left) and KS statistic-based benchmarking standard (right). Query signature was generated based on LIRI cohort. (**C**) Visualization of AUC-based (left) and KS statistic-based (right) performance measurements of XSum method on standardized data for discerning the optimal operating parameter. (**D**) Diagram summarizing the workflow and the important components associated with the investigation process of the optimal query signature size. (**E**) Relationship between query signature size determined by iterative fold change-based approach and retrieval performance evaluated by AUC-based standard (left) and KS statistic-based standard (right). (**F**) Relationship between query signature size determined by random sampling-based approach and retrieval performance evaluated by AUC-based standard (left) and KS statistic-based standard (right). LOESS polynomial regression analysis was performed for curve fitting. AUC, area under the curve; KS, Kolmogorov-Smirnov.

The two independent benchmarking standards enabled us to quantitatively assess the retrieval performance of different signatures matching methods. Six available methods, including eXtreme Sum (XSum) (Cheng et al., 2014), eXtreme Cosine (XCos) (Cheng et al., 2013; Cheng et al., 2014), eXtreme Pearson (XCor) (Zhou et al., 2018), eXtreme Spearman (XSpe) (Zhou et al., 2018), KS test (Lamb et al., 2006), and the Reverse Gene Expression Score (RGES) (Chen et al., 2017a), were included for performance comparison. To minimize technical bias introduced by different query signatures, four HCC signatures with different sizes generated from distinct data sets were utilized for benchmarking (Supplementary file 5). Of these, Sig_gastro_ (Chen et al., 2017b) and Sig_NC_ (Chen et al., 2017a) were directly obtained from previous publications, while Sig_LIRI_ and Sig_GSE54236_ were generated using RNA-seq data from LIRI cohort and microarray data from GSE54236, respectively. A brief summary of the above essential components involved in the evaluation process was presented in Figure 4A.

**Figure 4—figure supplement 1.**
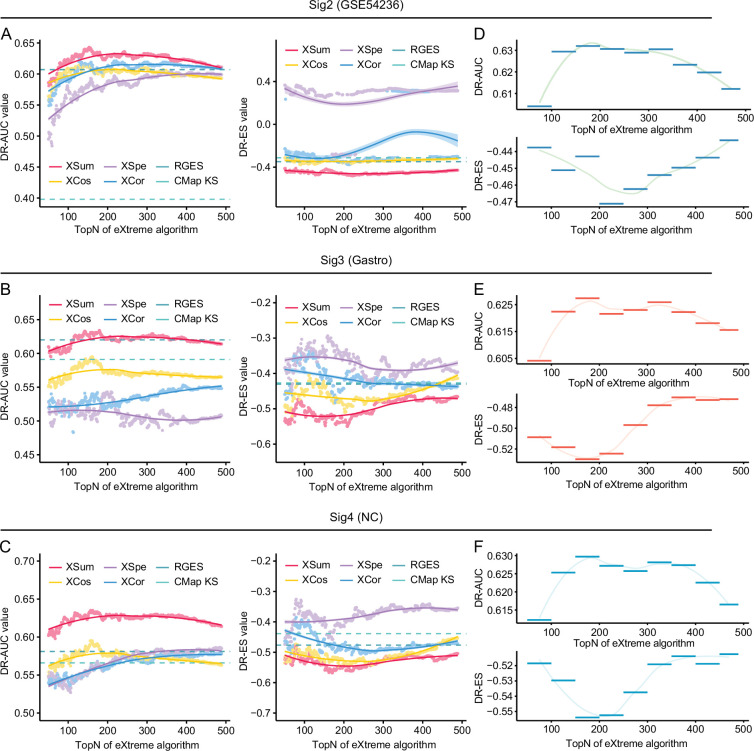
Benchmarking methodologies and parameters in the conditions of using different query signatures. Retrieval performance of six matching methods evaluated by AUC-based benchmarking standard (left) and KS statistic-based benchmarking standard (right) in the conditions of using SigGSE54236 (**A**), Sig_gastro_ (**B**), and Sig_NC_ (**C**) for querying LINCS. AUC-based (upper) and KS statistic-based (lower) standardized performance measurements of XSum method in the conditions of using SigGSE54236 (**D**), Sig_gastro_ (**E**), and Sig_NC_ (**F**) as query signatures. AUC, area under the curve; KS, Kolmogorov-Smirnov.

**Figure 4—figure supplement 2.**
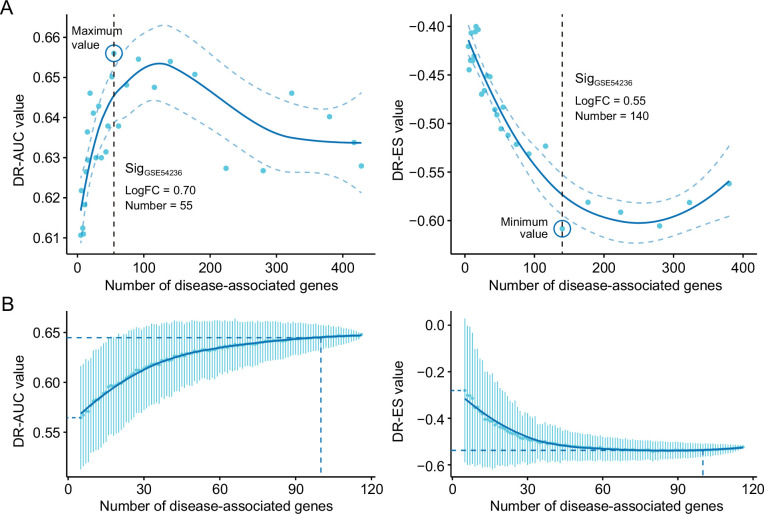
The influences of query signature size on retrieval performance. Relationship between query signature size determined by iterative fold change-based approach (**A**) or random sampling-based approach (**B**) and retrieval performance evaluated by AUC-based standard (left) and KS statistic-based standard (right). GSE54236 cohort was used for generating candidate query signatures for evaluation. AUC, area under the curve; KS, Kolmogorov-Smirnov.

Considering that the performance of eXtreme methods (including XSum, XCos, XCor, and XSpe) may be affected by the number of top genes (topN), we thus calculated the DR-AUC or DR-ES values of each eXtreme methods iteratively, using topN ranging from 50 to 489. In the condition of using Sig_LIRI_ as query signature, both benchmarking standards demonstrated that XSum outperformed other five methods across almost all candidate topNs (Figure 4B). Concordantly, when using other three query signatures, XSum also achieved better performance compared with other methods, except in the case of using Sig_Gastro_ as query signature and AUC-based standard for benchmarking, where RGES showed a similar performance with XSum (Figure 4—figure supplement 1A-C). Generally, XSum exhibited a consistently excellent performance, independently of the query signature and benchmarking standard (Supplementary file 6A and B). In addition, our analyses also demonstrated that the recently developed RGES (a modification of the KS method) was superior to the KS method and might serve as an alternative approach for KS-based connectivity mapping (Chen et al., 2017a).

We next sought to find the most appropriate topN value for applying XSum method to achieve the best retrieval performance. Directly selecting the exact topN value where corresponding DR-AUC/DR-ES reached their maximum/minimum might cause bias and could be prone to overfitting. Given the continuous trait of candidate topNs, we chose to divide them into several smaller bins, typically 50 topN values in each bin. The DR-AUC/DR-ES of a given bin was defined as the mean DR-AUC/DR-ES values within this bin, which could decrease potential influences brought about by outliers. With this normalization approach, relatively consistent results across varying conditions were obtained. The optimal window with the best performance was either ‘top150–200’ or ‘top200–250’ (Supplementary file 6C). Besides, we also observed a biphasic pattern of fitting curves, with the inflection points appearing where topNs were around 200 (Figure 4C, Figure 4—figure supplement 1D-F). Collectively, we supposed that topN of 200 could serve as a rough guide.

### A query signature size of 100 is applicable for drug retrieval

Next, we intended to further discern the optimal query signature size. Considering that Sig_gastro_ (N_gene_=44) and Sig_NC_ (N_gene_=73) had fixed and relatively small signature sizes, only signatures generated from LIRI and GSE54236 cohorts were utilized for the following investigation. We adopted two complementary approaches: (i) iterative fold change-based and (ii) random sampling-based approaches, to obtain query signatures with varying sizes (Figure 4D). The iterative fold change-based approach could create a number of signatures with discontinuous sizes through setting iterative threshold values of fold changes. The exact sizes of optimal signatures identified by this approach varied substantially (including 55, 79, 140, and 167). Despite this, similar trends of biphasic pattern with inflection points at around 100 under different conditions could still be observed (Figure 4E, Figure 4—figure supplement 2A). The approach based on random sampling was adopted as a complement. The results showed that, as the signature sizes increased, the DR-AUC/DR-ES values also increased/decreased and eventually converged when the signature size was more than 100 (Figure 4F, Figure 4—figure supplement 2B). Accordingly, we considered that a signature size of 100 could be selected as a good compromise. This conclusion remained valid in the conditions when other topN values were applied, such as 100 and 400 (results not shown).

### A good query signature should comprehensively reflect the clinical characteristics of corresponding disease

**Figure 5.**
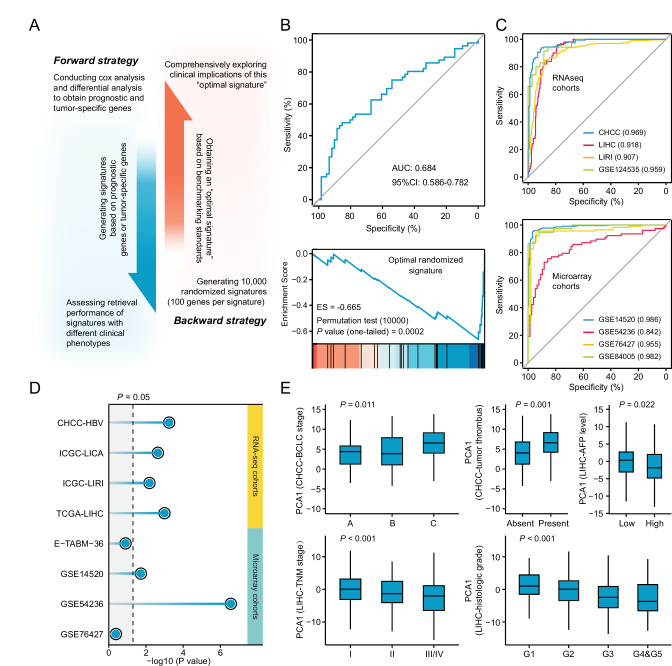
Necessary properties of a good query signatures. (**A**) Schematic illustration of forward and backward strategy adopted to investigate whether the factor associated with clinical phenotype of query signature can affect computational therapeutic discovery. (**B**) The DR-AUC value and DR-ES value of the optimal randomized signature showed by ROC curve (upper) and enrichment plot (lower). (**C**) The association between the optimal signature and the clinical phenotype of discordant expression pattern suggested by ROC curves based on RNA sequencing cohorts (upper) and Microarray cohorts (lower). (**D**) The association between the optimal signature and the clinical phenotype of prognosis. Color toward gray indicates no statistical significance. (**E**) The association between the optimal signature and multiple clinical features, including BCLC and TNM stage, tumor thrombus, AFP level, and histologic grade. Data are presented as median±quartiles. N≥100. Statistical significance of difference between groups was determined using either Kruskal-Wallis or Wilcoxon sum rank tests.

Many previous studies chose to compare normal versus diseased states to define disease signatures. However, signatures that are generated based on other clinical phenotypes, such as prognosis and metastasis, can also be used to query LINCS. Aiming to figure out whether this factor could also affect the performance of drug retrieval, we designed a forward and a backward strategy (Figure 5A). The application of forward strategy was based on two types of signatures, general signatures (representing discordant expression pattern between normal and tumor tissues) and prognostic signatures (associated with survival outcomes). We compared the above two signature phenotypes across varied signature sizes. Unfortunately, the results under different data sets and benchmarking standards were highly inconsistent. This strategy thus failed to provide a definitive conclusion (Figure 5—figure supplement 1A and B).

**Figure 5—figure supplement 1.**
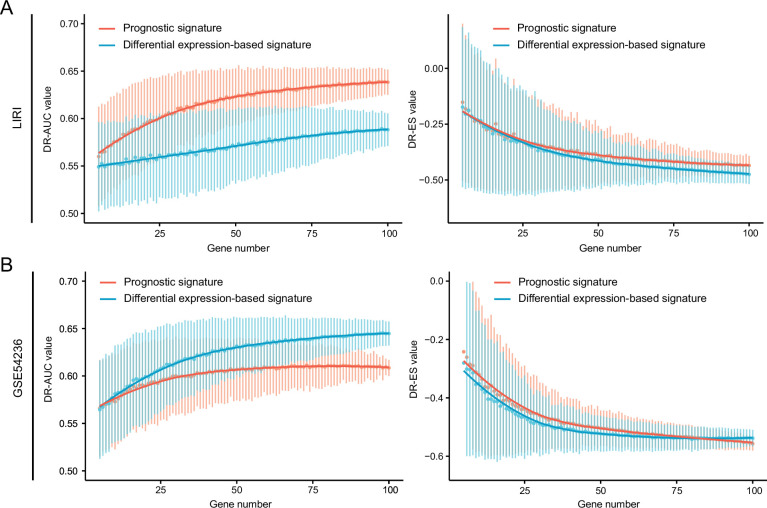
The influences of query signature phenotype on retrieval performance. Comparison of retrieval performance evaluated by AUC-based standard (left) and KS statistic-based standard (right) between prognosis-associated signatures and discordant expression-associated signatures generated by LIRI cohort (**A**) or GSE54236 cohort (**B**). AUC, area under the curve; KS, Kolmogorov-Smirnov.

Opposed to the forward strategy, backward strategy started from creating a collection of 10,000 random signatures, followed by determining the optimal signature for clinical implication evaluation. The optimal random signature was determined according to both benchmarking standards. Exploring the clinical values of this signature might reveal some necessary features possessed by a ‘good’ query signature (Figure 5B). A comprehensive clinical evaluation on the optimal signature was carried out based on five RNA-seq and five microarray clinical cohorts from three perspectives. First, the ability of this signature to distinguish tumors from non-tumors was investigated. Briefly, we extracted the first principal components (PC1) of this signature to represent its overall expression pattern. AUC was used here as a measurement of the classification capability. The results showed that more than 0.90 of AUC can be obtained in seven out of the eight cohorts (87.5%), indicating that the ability to discern the difference between diseased and normal states might be an indispensable property for achieving good retrieval performance (Figure 5C). Next, we intended to find out whether the optimal signature should be a prognostic indicator. Cox regression analyses were conducted to investigate the association between the signature expression (PC1) and clinical outcome. As a result, significant prognostic implications of the optimal signature could be observed in six out of the eight cohorts (75%), suggesting that prognostic significance was also a necessary characteristic (Figure 5D). At last, the association between signature expression and other clinical features was explored. Considering that CHCC and LIHC cohorts held the most abundant clinical information, corresponding analyses were thus conducted on these two cohorts. The results showed that there was a significant correlation between the optimal signature and multiple clinical features, including BCLC stage (p=0.011), tumor thrombus (p=0.001), AFP level (p=0.022), TNM stage (p<0.001), and histologic grade (p<0.001) (Figure 5E). Accordingly, we concluded that a good query signature should possess the ability to comprehensively recapitulate the clinical features of corresponding disease, rather than only reflect the disease characteristic from single perspective.

### Generation of novel liver cancer signature

**Figure 6.**
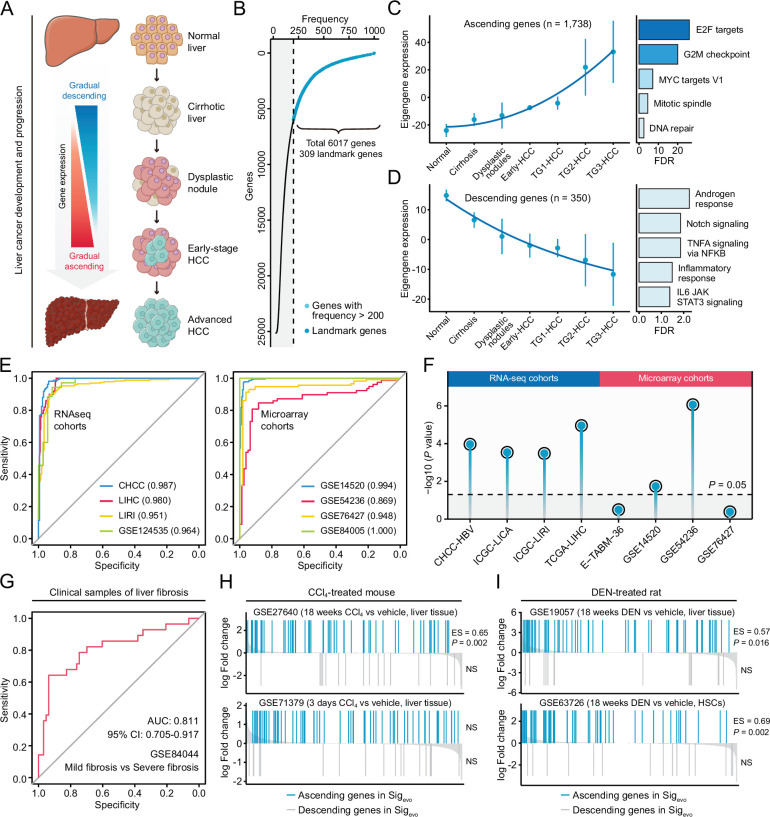
Development of a novel signature representing the initiation and progression of liver cancer. (**A**) Schematic of the stepwise process of liver cancer initiation and progression. (**B**) Preliminary screening of developmental stage-associated genes by random forests algorithm based on GSE89377. (**C**) The expression pattern of the ‘ascending’ module discerned by WGCNA analysis (left) and the enriched biological processes determined by hypergeometric test (right). (**D**) The expression pattern of the ‘descending’ module (left) and the enriched biological processes (right). (**E**) The performance evaluation of the Sig_evo_ for discerning the difference between tumor and normal tissues based on RNA sequencing cohorts (left) and microarray cohorts (right). (**F**) The association between the Sig_evo_ and the clinical phenotype of prognosis. Color toward gray indicates no statistical significance. (**G**) The association between the Sig_evo_ and fibrosis-related phenotype suggested by ROC curve. (**H**) The association between Sig_evo_ and CCl4-induced expression changes in liver tissues of mice. The enrichment scores and statistical significance were determined by gene set enrichment analysis. (**I**) The association between Sig_evo_ and DEN-induced expression changes in liver tissues of rats. WGCNA, weighted gene co-expression network analysis.

The conclusions from the above analyses were then applied to establish a signature representing liver cancer initiation and development, which could be utilized to query compounds with potential therapeutic as well as preventive effects against liver cancer. The generation of this evolution-associated signature was based on the concept that the initiation and progression of liver cancer was a stepwise process with gradually acquired advantageous biological capabilities (Figure 6A). Therefore, conceptually, antagonizing genes that were most related to these stages could be a potential therapeutic strategy. Through implementing random forests algorithm on GSE89377 cohort, preliminary screening was performed to include stage-associated genes, where genes with greater predictive power were selected for further analysis. This screening yielded a total of 6017 stage-associated genes (23.9%), of which 309 were landmark genes (Figure 6B). Next, we conducted weighted gene co-expression network analysis (WGCNA) to obtain co-expressed modules with diverse expression patterns (Figure 6—figure supplement 1A). Seven gene modules were discerned by WGCNA analysis (Figure 6—figure supplement 1B and C), and two of them, which we termed the ‘ascending’ module (N=1738) and the ‘descending’ module (N=350) for their greatest relevance to stages and patterns of linear evolution from normal to cancer, were retained for further analyses (Figure 6C and D). Biological processes associated with genes in these two modules were investigated. We found that the ‘ascending’ module was closely associated with proliferation (Figure 6C), while the ‘descending’ module was enriched in several different types of processes (Figure 6D). There were 159 genes in common between these two modules and landmarks. Based on the aforementioned recommendation of query signature size, we sought to further reduce the size of 159–100. This procedure was carried out using HCC occurrence-related clinical and molecular data from GSE15654 cohort. In brief, 10,000 random signatures, each containing 100 genes, were generated based on the 159-gene panel. The one which had the most significant association with HCC occurrence was considered as the optimal query signature (Figure 6—figure supplement 2A and B). This analysis yielded a signature comprised of 82 ascending genes and 18 descending genes, which was then named as Sig_evo_ (Supplementary file 7). The linear evolution pattern of Sig_evo_ remained present in training (Figure 6—figure supplement 2C) as well as an independent validation cohort (Figure 6—figure supplement 2D).

**Figure 6—figure supplement 1.**
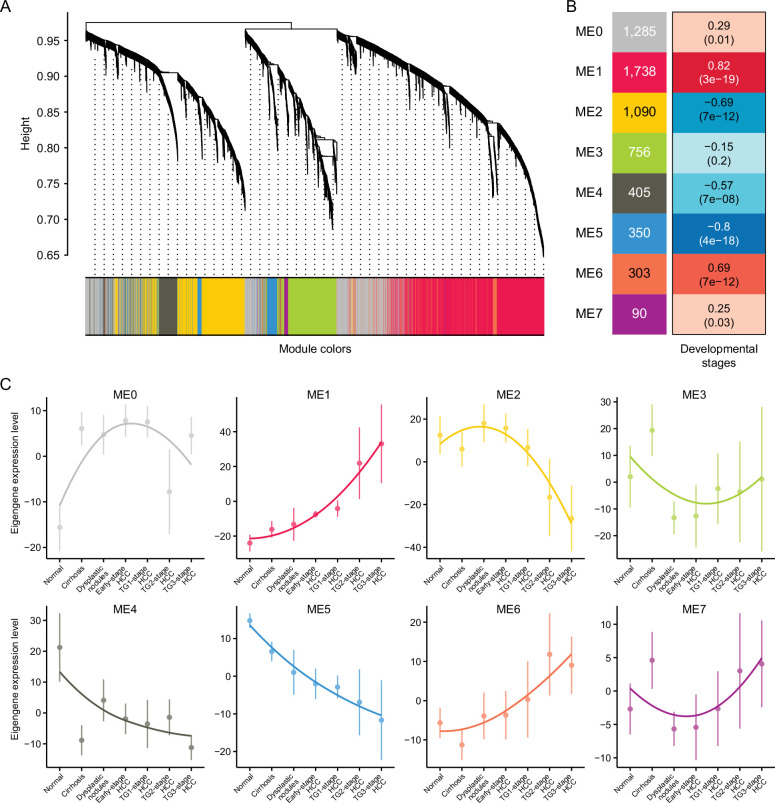
Weighted gene co-expression network analysis (WGCNA). (**A**) Hierarchical cluster tree showing eight modules of co-expressed genes identified by WGCNA. Each of the stage-associated genes is represented by a leaf in the tree, and each of the eight modules by a major tree branch. (**B**) Module-trait (developmental stage) relationship and corresponding p values. The left panel shows the eight modules and the number of genes in each module, and the color scale on right shows module-trait correlation from –1 (blue) to 1 (red). (**C**) The expression pattern of eight modules. Note that gray module represents unassigned genes.

**Figure 6—figure supplement 2.**
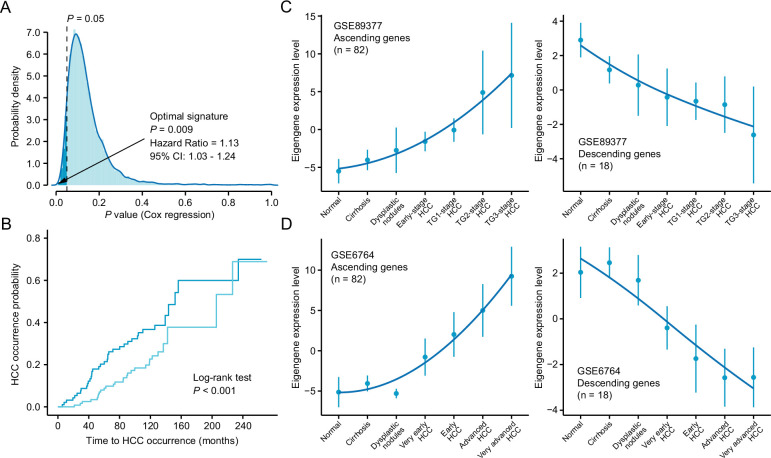
Identification and validation of the novel query signature. (**A**) The distribution of the statistical significance results of 10,000 cox proportional hazards regression analysis. The optimal signature with the most significant relevance to HCC occurrence was labeled in the figure. (**B**) Kaplan-Meier cumulative hazard rates for HCC occurrence according to the groups determined by the optimal signature expression. (**C**) Validation of the expression pattern of the ‘ascending’ (left) and ‘descending’ (right) module based on the optimal signature in GSE89377 cohort. (**D**) Validation of the expression pattern of the ‘ascending’ (left) and ‘descending’ (right) module based on the optimal signature in GSE6764 cohort. HCC, hepatocellular carcinoma.

As previously discussed, a good query signature should reflect the clinical features of corresponding disease comprehensively. Therefore, we systematically surveyed the association between Sig_evo_ and the clinical phenotypes of precancerous/cancerous liver lesions using clinical and experimental data from both human and animal data sets. First, based on clinical cohorts of HCC, we demonstrated that Sig_evo_ had a remarkable capability for distinguishing tumors from non-tumors, with a median AUC of 0.972 in all eight cohorts (Figure 6E). Besides, this signature also held great prognostic power in HCC, as indicated by the results of Cox analyses (Figure 6F). Next, in view of the crucial role of fibrosis in driving hepatocarcinogenesis, further investigation was performed to validate its relevance to fibrosis-related phenotype. The result suggested that Sig_evo_ could also effectively differentiate between mild (S0/S1) and severe (S3/S4) fibrosis (Figure 6G). Additionally, we collected four experimental data sets, including two carbon tetrachloride (CCl_4_)-treated mouse data sets and two diethylnitrosamine (DEN)-treated rat data sets, to assess the enrichment levels of Sig_evo_ in mouse and rat fibrosis models. It could be observed that ascending genes in Sig_evo_ were significantly enriched in both CCl_4_-treated (GSE27640) and DEN-treated (GSE19057) liver tissues (Figure 6H and I). However, descending genes did not exhibit any significant enrichment pattern in all included data sets, possibly due to the limited gene number (Figure 6H, I). Notably, the expression profiles in GSE63726 were derived from non-parenchymal cell fractions which had abundant hepatic stellate cells (HSCs), and thus the significant enrichment could provide the evidence that this signature might reflect the molecular feature of HSC activation (Figure 6I). In summary, the Sig_evo_ fully complied with the criteria of a good query signature and was then employed for querying LINCS.

**Figure 7.**
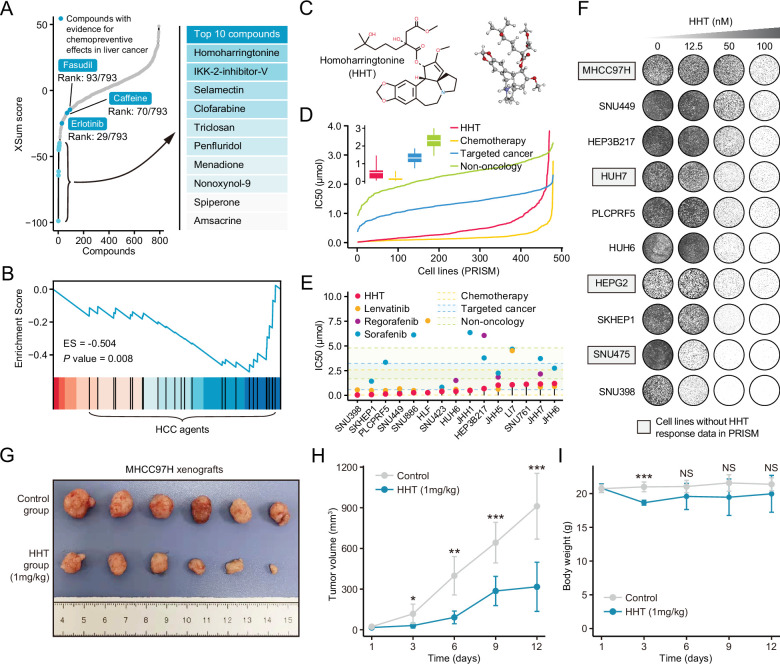
Homoharringtonine (HHT) has significant tumor killing activity both in vitro and in vivo. (**A**) Results of best practice approach-based computational dr_evo_ as query signature. Top ranked 10 compounds with highest reversal potency were illustrated in the right panel. (**B**) Enrichment of HCC agents in compounds with reversal potency (XSum score<0). Statistical significance was determined based on the null distribution formed by 10,000 permutations. (**C**) 2D (left) and 3D (right) chemical structure of HHT. (**D**) Comparison of distribution of compound activity between HHT and three different drug categories, including chemotherapy (N=45 compounds), targeted cancer agents (N=419 compounds), and non-oncology (N=362 compounds). The IC_50_ values (from PRISM data set) of each drug category in each cell line (N=482) were determined through calculating the median IC_50_ value across all the compounds in this category. Data are presented as median±quartiles, N≥100. (**E**) The drug sensitivity data of HHT (achieved from PRISM data set) across liver cancer cell lines. The drug sensitivities of two HCC agents in the first-line (sorafenib and lenvatinib) and one HCC agent in the second-line (regorafenib) were also presented for comparison. Areas with different colors denote the interquartile range of median IC_50_ values of compounds within different drug categories. (**F**) Long-term cell proliferation assay for testing the anti-tumor activity of HHT across 10 liver cancer cell lines. Of these, four cell lines have not been profiled by PRISM for the sensitivity to HHT. (**G**) Macroscopic image of tumors harvested from xenograft mice treated with vehicle (upper) and HHT (lower). (**H**) Longitudinal tumor volume progression of subcutaneous MHCC97H xenograft tumors treated with vehicle (N=6) and HHT (N=6). The statistical significance of difference between groups was determined using Student’s t-test. Data are represented as mean ± SD. (**I**) Body weight changes of mice in control (N=6) and HHT-treated (N=6) groups. Statistical significance was determined using Student’s t-test. Data are represented as mean ± SD. *p<0.05, **p<0.01, ***p<0.001. NS, not significant. HCC, hepatocellular carcinoma. Figure 7—source data 1.Drug-induced expression changes across different cell lines as well as different concentrations.

Using the optimal method (XSum) and a compromising parameter (topN=200), we matched Sig_evo_ with HepG2-derived compound signatures in LINCS and obtained the similarity scores of all compounds (lower scores implied higher reversal potency and greater potential for application). After excluding preclinical agents or agents withdrawn from the market, 793 agents remained (Corsello et al., 2017). These agents were then considered repositioning candidates (Supplementary file 8). Interestingly, some agents which were previously proved to have chemopreventive effects, including erlotinib (Fuchs et al., 2014), caffeine (Hoshida et al., 2012), and fasudil (Nakagawa et al., 2016), dominated relatively high rankings on the list (Figure 7A). Besides, anti-HCC agents were also found to be enriched significantly in compounds with reversal potency (Figure 7B). These findings collectively supported the reliability of the prediction results.

**Figure 7—figure supplement 1.**
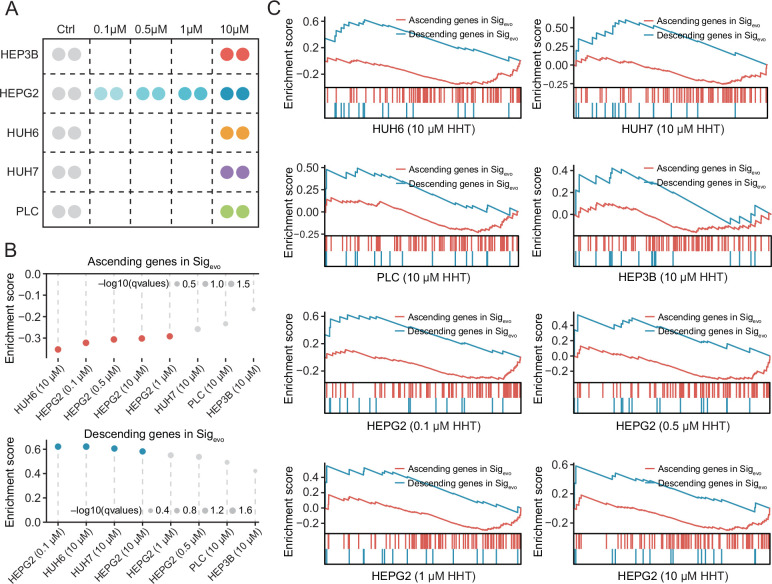
Reversal effect of HHT on Sig_evo_ across different conditions. (**A**) Schematic figure illustrating the experimental design. (**B**) Enrichment scores of ascending (upper) and descending (lower) gene sets in the condition of different cell types as well as different concentrations. A positive enrichment score of a given gene set indicates the enrichment of HHT-induced upregulated genes and a negative enrichment score indicates the enrichment of HHT-induced downregulated genes. Color toward gray indicates no statistical significance. (**C**) GSEA plots of ascending and descending gene sets in different conditions. HHT, homoharringtonine.

**Figure 7—figure supplement 2.**
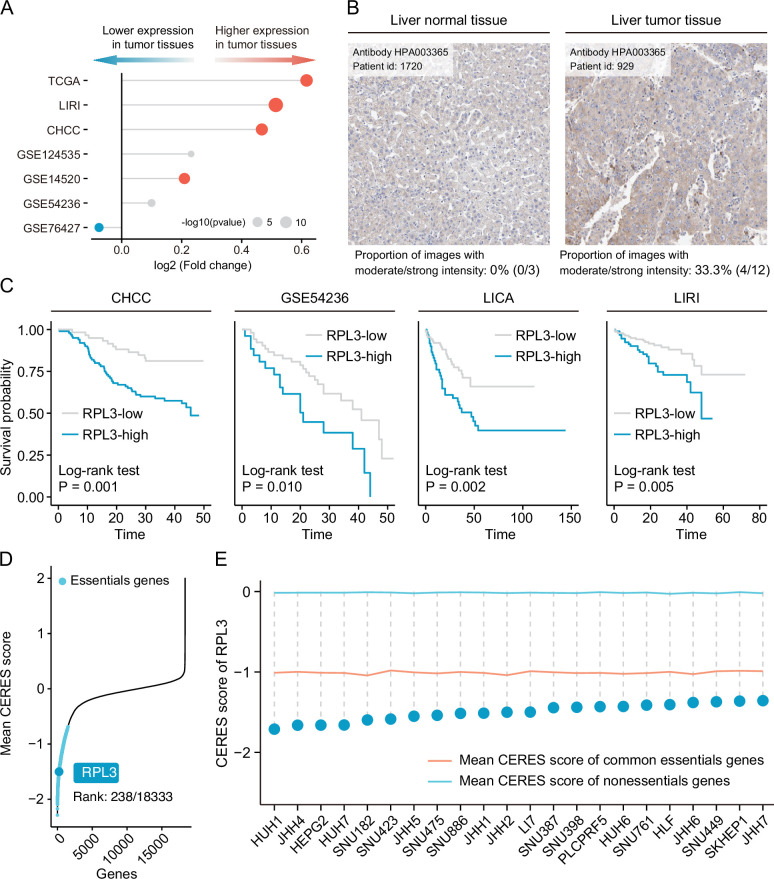
Clinical and biological characterization of RPL3 in liver cancer. (**A**) The comparison of mRNA expression level of RPL3 between tumor and non-tumor tissues across seven HCC clinical cohorts. Statistical significance of difference was determined using Wilcoxon rank-sum test. Color toward gray indicates no statistical significance. (**B**) Representative images of immunohistochemical staining of RPL3 in liver normal (left) and tumor tissues (right) from the Human Protein Atlas (HPA) program. (**C**) Comparison of survival curves between high RPL3 expression and low RPL3 expression groups. (**D**) Distribution of gene dependency score (CERES score) of 18,333 protein-coding genes in liver cancer cell lines. A lower CERES score of certain gene indicates a higher likelihood that this gene is essential in cell growth and survival. (**E**) The gene dependency of RPL3 across 22 liver cancer cell lines.

**Figure 7—figure supplement 3.**
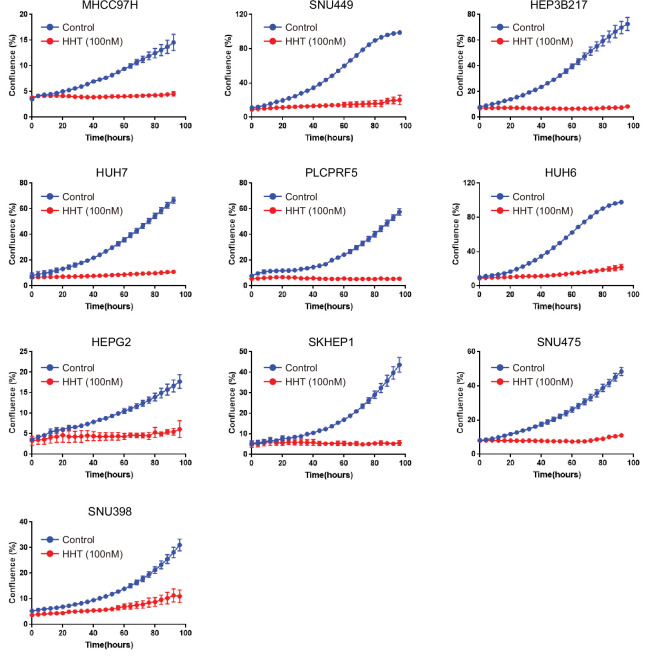
The effect of HHT on cell proliferation across 10 liver cancer cell lines. Cell proliferation rate was analyzed by IncuCyte ZOOM system every 4 hr for 100 hr. HHT, homoharringtonine.

**Figure 7—figure supplement 4.**
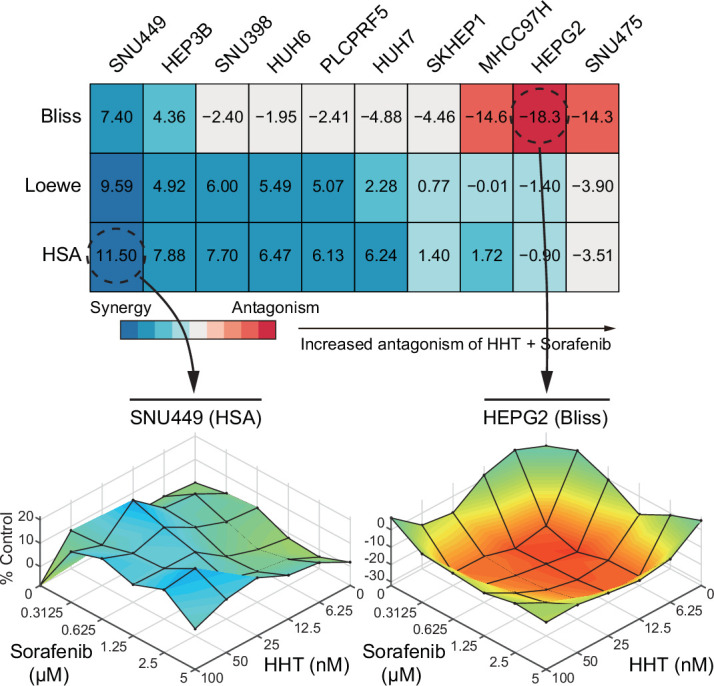
Summary of the anti-tumor effect of HHT-sorafenib combination across 10 liver cancer cell lines. Heatmap represents the summary of the synergistic effects of HHT-sorafenib combination. Higher score indicates greater synergism. The lower panel displays two representative surface plots that show the overall synergistic (left) and antagonistic (right) effects of the combination treatments. HHT, homoharringtonine.

**Figure 7—figure supplement 5.**
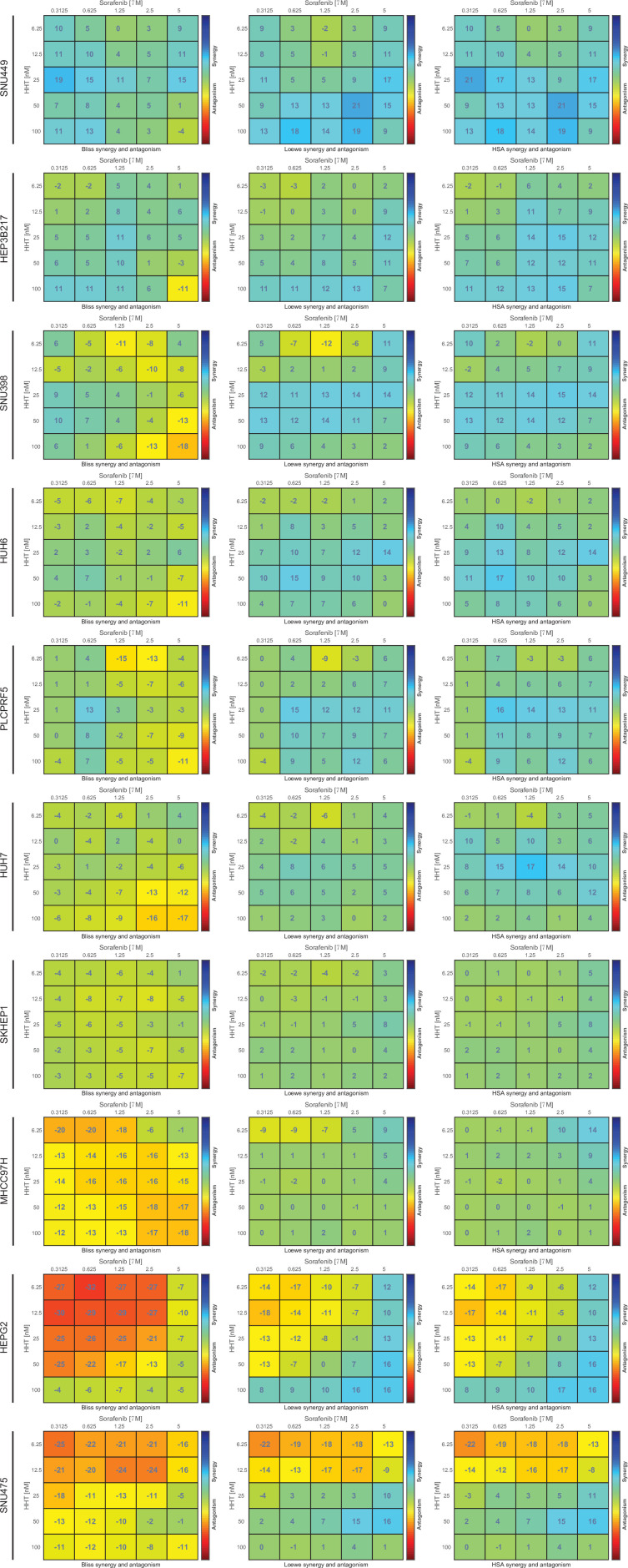
Detailed information of the anti-tumor effect of HHT-sorafenib combination across 10 liver cancer cell lines. Combinational effects were measured using three models, including Bliss independence model, Loewe additivity model, and Highest single agent (HAS) model. HHT, homoharringtonine.

### Homoharringtonine is a candidate anti-liver agent

According to the computational results, homoharringtonine (HHT) (Figure 7C), a protein synthesis inhibitor targeting RPL3, had the highest reversal potency among 793 repositioning candidates (Tujebajeva et al., 1989). To prove that the reversal effect of HHT is not cell type- or concertation-dependent, we generated HHT-perturbed expression data using five different liver cancer cell lines (Hep3B, HepG2, Huh6, Huh7, and PLC) and four different concentrations (0.1 μM, 0.5 μM, 1 μM, and 10 μM). HHT with a fixed concentration of 10 μM (a standard concentration in CMap and LINCS) was used to treat different cell lines and a single cell line HepG2 (a cell line used in LINCS) was perturbed by HHT with varying concentrations (Figure 7—figure supplement 1A). HHT signatures were obtained through calculating the fold changes of HHT-treated samples to control samples. Subsequently, GSEA was conducted against different HHT signatures, taking ascending and descending genes in Sig_evo_ as query gene sets separately. The results indicated that the ascending genes tended to enrich in HHT-induced downregulated genes (ES<0), while descending genes appeared to be more associated with HHT-induced upregulated genes (ES>0), suggesting that the ability of HHT to reverse the Sig_evo_ was independent of cell type and treatment concentration (Figure 7—figure supplement 1B and C, Figure 7—source data 1, GSE193897).

As the drug target of HHT, RPL3 was characterized for its clinical and biological implications. Comprehensive comparisons of the expression of RPL3 between tumor and non-tumor tissues were conducted using seven clinical cohorts with available expression profiles of both tumor and non-tumor tissues. The results showed that RPL3 had higher expression levels in tumor compared with non-tumor tissues in more than half the clinical cohorts (57.1%) (Figure 7—figure supplement 2A). The increase of protein expression of RPL3 could also be observed in tumor tissues (Figure 7—figure supplement 2B), as shown by immunohistochemical images from the Human Protein Atlas (Uhlén et al., 2015). Higher expression of RPL3 also indicated worse prognosis (Figure 7—figure supplement 2C). In addition, leveraging CRISPR-based screening data from Project Achilles (Meyers et al., 2017), we found that RPL3 was essential for maintaining the survival and growth of all liver cancer cell lines (Figure 7—figure supplement 2D, E). Above results demonstrated the rationality of RPL3 inhibition for treating liver cancer.

### Homoharringtonine has a remarkable therapeutic effect against liver cancer

To investigate the in vitro anti-tumor activity of HHT against liver cancer, we analyzed the drug response data of HHT from PRISM data set (Corsello et al., 2020). It could be observed that HHT had a lower distribution of IC_50_ values across 482 PRISM cell lines compared with molecular-targeted agents and non-oncology agents (Figure 7D). Of note, in liver cancer cell lines, HHT exhibited a powerful tumor suppressor activity with a median IC_50_ value of 0.408 μM, which was numerically lower than that of other three Food and Drug Administration (FDA)-approved HCC agents (lenvatinib: 0.617 μM; regorafenib: 2.009 μM; sorafenib: 3.348 μM) (Figure 7E). The in vitro anti-tumor activity of HHT was corroborated by the long-term cell proliferation assay (Figure 7F) and short-term IncuCyte real-time assay (Figure 7—figure supplement 3). In addition, in vivo efficacy of HHT was also evaluated using subcutaneous xenograft model of MHCC97H cell line. The result demonstrated that HHT could significantly inhibit the growth of xenograft tumors (Figure 7G and H), with limited drug-related toxicity (Figure 7I). Since co-administration of HHT with other approved agents was more likely to have clinical significance, we also interrogated whether HHT could augment the tumor-killing effect of sorafenib. Three different statistical models were adopted for synergy estimation. The results suggested that HHT could indeed synergize with sorafenib in many conditions, albeit not very remarkable in general (Figure 7—figure supplements 4 and 5).

### Homoharringtonine treatment can alleviate liver fibrosis both in vivo and in vitro

**Figure 8.**
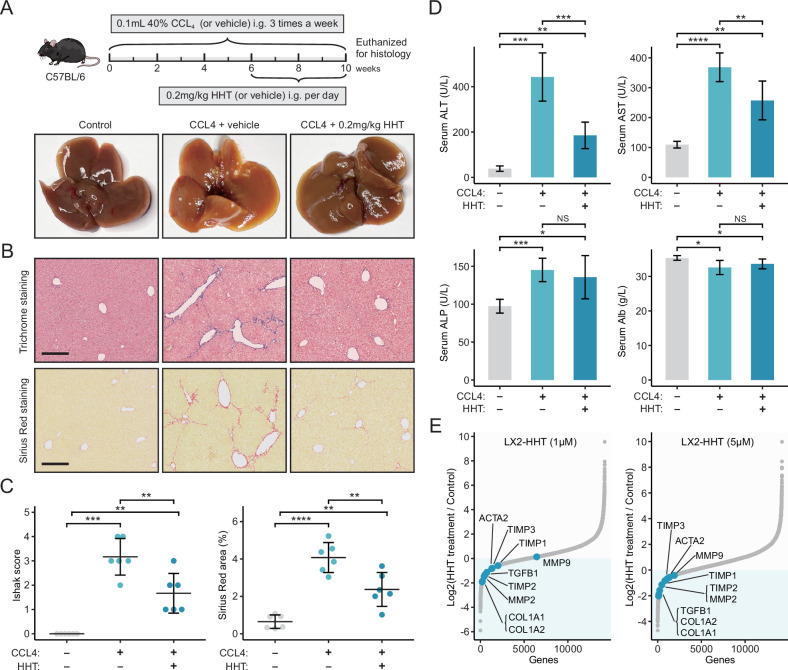
HHT has significant in vivo anti-fibrotic effects. (**A**) Schematic diagram (upper) of the experimental design for validating the anti-fibrotic ability of HHT and representative photographs (lower) of the livers harvested from different groups at the time of sacrifice. (**B**) Representative images of Masson’s trichrome staining and Sirius Red staining of liver tissues from different groups (scale bars: 250 µm). (**C**) Comparisons of Ishak scores (left) and Sirius Red-based collagen quantification (right) between different groups. Statistical significance was determined using one-way ANOVA followed by Tukey multiple comparison test. Data are represented as mean ± SD (N=6 in each group). (**D**) Comparisons of serum levels of ALT, AST, ALP, and Alb between different groups. Statistical significance was determined using one-way ANOVA followed by Tukey multiple comparison test. Data are represented as mean ± SD (N=6 in each group). (**E**) Differential expression of nine fibrosis-associated genes between HHT-treated and HHT-untreated LX-2 cells. *p<0.05, **p<0.01, ***p<0.001, ****p<0.0001. HHT, homoharringtonine; NS, not significant. Figure 8—source data 1.Sequencing results of HHT-treated LX2 cells. Figure 8—source data 2.Raw unedited plots.

Liver fibrosis occurs when the liver tissue is repeatedly and continuously injured, which is a crucial risk factor for hepatocarcinogenesis (O’Rourke et al., 2018). Since we have proved that Sig_evo_ was associated with liver fibrosis using clinical and animal-derived data, it could be postulated that HHT also had the potential to alleviate liver fibrosis. The antifibrotic effect of HHT was first assessed using carbon tetrachloride (CCL_4_)-induced mouse liver fibrosis model (Figure 8A). The results suggested that HHT could significantly reduce Ishak scores and positive area of Sirius Red staining compared to vehicle controls (Figure 8B and C). Besides, HHT treatment could also lead to significant reduction of serum levels of alanine transaminase (ALT) and aspartate transaminase (AST) (Figure 8D). These observations demonstrated that HHT can impede fibrosis development and partially rescued hepatic function in CCL_4_-induced mouse model. The activation of HSCs is one of the key steps in fibrosis development (Zhou et al., 2014). To determine whether HHT could inhibit the activation of HSCs, in vitro experiments based on TGF-β1-activated human HSC line LX-2 were further conducted. LX-2 cells were treated with vehicle or HHT (1 μM and 5 μM) for 6 hr, followed by RNA-seq for quantifying HHT-induced expression changes. Nine fibrotic genes from previous publications were collected; high expression level of these genes represented the activation status of HSCs. After HHT treatments, the expression level of almost all fibrotic genes was downregulated (Figure 8E, Figure 8—figure supplement 1, Figure 8—source data 1, GSE180243). The downregulated tendency of the two most critical genes which encoded collagen I and α-SMA were further corroborated by the quantitative real-time PCR (Figure 8—figure supplement 2A). Additionally, the protein-level expression of collagen I and α-SMA was also detected using western blot (Figure 8—figure supplement 2B, Figure 8—source data 2) and immunofluorescence (Figure 8—figure supplement 2C). The results showed that HHT could inhibit the protein expression of collagen I and α-SMA as well. Taken collectively, HHT can inhibit the progression of liver fibrosis via suppressing HSC activation and thus may have certain preventive effects on liver cancer.

**Figure 8—figure supplement 1.**
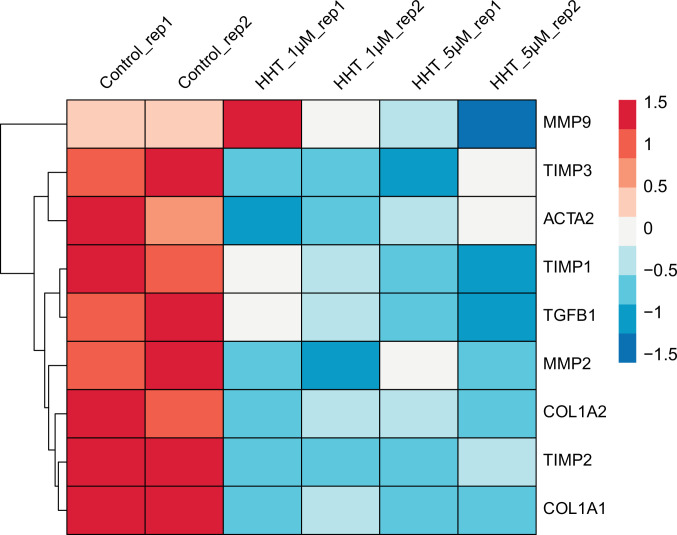
Comparison of the expression of nine fibrosis-associated genes between control LX2 and HHT-treated LX2. Gene expression was measured by RNA-seq and the processed data has been provided as the source data file.

**Figure 8—figure supplement 2.**
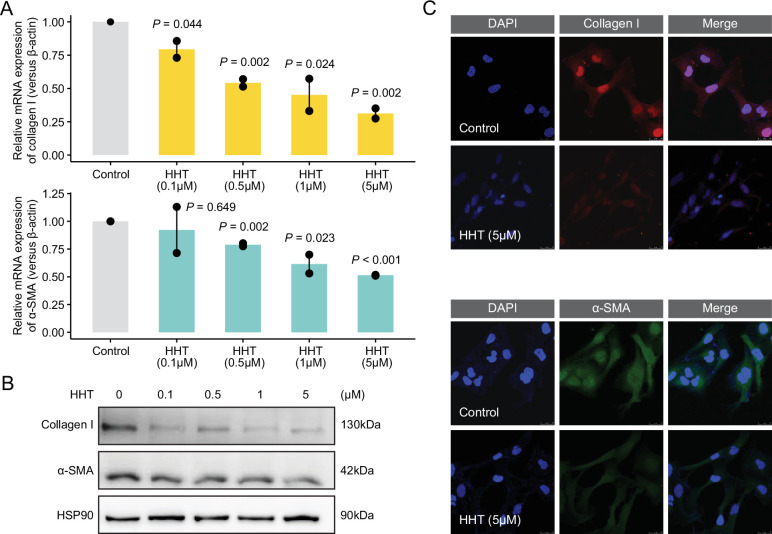
In vitro anti-fibrotic effects of HHT. (**A**) Quantitative real-time PCR-based mRNA expression level of collagen I (upper) and α-SMA (lower) of LX-2 cells treated with gradient concentrations of HHT for 6 hr. Statistical significance was determined using Student’s t-test. Data are represented as mean ± SD, N=3. (**B**) Western blot-based protein expression level of collagen I and α-SMA of LX-2 cells treated with gradient concentrations of HHT for 24 hr. (**C**) Representative images of immunofluorescence staining of LX-2 cells with antibody against collagen I (upper) and α-SMA (lower). HHT, homoharringtonine.

## Discussion

In recent years, the explosive growth of pharmacogenomic data enables the development of computational drug discovery and repositioning, leading to many remarkable findings of novel therapeutics (Kong et al., 2020; Stathias et al., 2018; Yang et al., 2021). Owing to the success of CMap and LINCS projects (Lamb et al., 2006; Subramanian et al., 2017), signature reversion-based computational drug discovery approach has been extensively used (Chen et al., 2017a; Chen et al., 2017b; Dudley et al., 2011; van Noort et al., 2014; Wei et al., 2006). However, lack of suitable benchmarking standards for evaluating drug repositioning performance limits further improvement of this approach. Some studies proposed that the benchmarks assessing drug-drug similarity, such as the anatomical therapeutic chemical (ATC) system, could be taken as alternative standards to indirectly determine the optimal methodologies and parameters of computational repositioning (Cheng et al., 2013; Zhou et al., 2018). However, considering the great difference between the two situations, developing tailored benchmarking standards for assessing disease-drug similarity would be more desirable (Cheng et al., 2014). In this study, we proposed two novel benchmarking standards, AUC-based and KS statistic-based standards. Despite being mutually independent, the evaluation results of the two standards were highly consistent, demonstrating their rationality and robustness.

These two standards enable the establishment of a standardized procedure for performing more effective signature-based drug prediction. We first determined that using reference signatures from one of the most relevant cell lines with the disease of interest instead of from a non-touchstone cell line or aggregation-based consensus results was a preferable option to exploit LINCS data. Next, XSum was identified as an optimal method for matching compound and disease signatures. Interestingly, a prior study that made a comparison of drug retrieval performance between XSum, XCos, and KS methods using a totally different benchmarking standard from ours also come to the same conclusion (Cheng et al., 2014). Furthermore, we also uncovered an appropriate parameter of XSum (topN=200), which lacked guidance previously.

Most of the current investigations and methodological developments were focused on reference signatures and signature matching methods. By contrast, relatively limited efforts have been made to standardize the generation of query signatures (Chan et al., 2019; Wen et al., 2016; Wen et al., 2015). In this study, two potential factors, signature phenotypes and signature size, were systematically analyzed. Through adopting two independent approaches, an appropriate query signature size of 100 was determined. However, prior studies considered a reduced number of 50 as the optimal size of query signatures (Chen et al., 2017a; Parkkinen and Kaski, 2014). It is reasonable to speculate that the utility of different signature matching methods (XSum in this study and KS-based methods in other studies) and also the different benchmarking standards may be responsible for the discrepancy. Next, we determined that a good query signature should hold the ability to comprehensively characterize the clinical features of corresponding disease. This finding seemed to be reasonable since disease was highly likely to be underrepresented when the query signature was generated based on a single clinical phenotype.

Based on these findings, we summarized the best practice approach for LINCS-based drug prediction. An application of this approach to liver cancer was then carried out. An evolution-associated query signature related to the development and progression of liver cancer was first constructed for drug retrieval. Following the best practice approach, HHT was identified as the candidate agent for its highest reversal potency. Since the query signature (Sig_evo_) could reflect the properties of liver cancer initiation and development, we considered that HHT might have both therapeutic and preventive effects on liver cancer. The therapeutic effect of HHT was assessed by in vitro cell line models as well as in vivo subcutaneous xenograft model. Both of them suggested remarkable tumor-killing activity of HHT. For validating the preventive effect, an indirect approach that focused on proving the anti-fibrotic effect of HHT was adopted. The results demonstrated that HHT could alleviate liver fibrosis in vivo and inhibit the activation of HSCs in vitro. Inhibition of liver fibrogenesis might prevent the progression of cirrhosis and thereby suppress HCC tumorigenesis (Fuchs et al., 2014). Therefore, we supposed that HHT had the potential to be taken as preventive agents for liver cancer as well. Notably, in view of the grim prognosis and imperfect treatment modalities of liver cancer, prevention of HCC development in patients at high risk of primary malignancy rather than treating patients at advanced stages is theoretically the most desirable approach to improve patient prognosis (Fujiwara et al., 2018; Nakagawa et al., 2016). As HHT has been approved by FDA for the treatment of chronic myelogenous leukemia, it can be tested directly in clinic without worrying about its safety problem (Kantarjian et al., 2013).

In this study, we have performed the most comprehensive surveys so far about the influencing factors of signature reversion-based drug prediction. Two novel benchmarking standards are proposed, providing new insight into the evaluation of related methodologies. All the findings in this study are verified independently by at least two different approaches, ensuring the reliability of the conclusions. Nevertheless, we also recognize several important limitations. First, with our design, our conclusions are conditional and hold only under the conditions of using compound profiles of HepG2 in LINCS as reference signatures. Further investigations using other LINCS data are required to extend current conclusions to other conditions. Second, the parameters recommended by us, including topN of 200 and query signature size of 100, are more or less based on our subjective judgments and should be taken as a rough guide. Although there are sufficient non-quantitative estimates supporting the use of these two parameters, more efforts are still needed to accurately determine the optimal parameters. Third, we only focused on analyzing the data from the project that utilized transcriptomic platforms to measure cell responses during perturbation experiments, and other omics data which are actively being generated by different LINCS centers might also be a good choice for computational drug discovery and repositioning (Keenan et al., 2018; Koleti et al., 2018). Recently, large-scale resources (CPPA) of perturbed protein responses have been generated (Zhao et al., 2020). Considering that proteins are the components of the basic functional units in biological pathways, investigating the optimal repositioning strategy based on proteomic resources may also have important implications.

In summary, our findings fill a knowledge gap in the area of LINCS-based computational repositioning. Through exploiting these findings, we also determined a promising anti-liver cancer agent HHT, of which the therapeutic and preventive effects have been validated experimentally.

## Materials and methods

**Key resources table:** 

Reagent type (species) or resource	Designation	Source or reference	Identifiers	Additional information
Cell line (*Homo sapiens*)	Hep3B	ATCC	Cat#: HB-8064;RRID:CVCL_0326	
Cell line (*H. sapiens*)	HepG2	ATCC	Cat#: HB-8065; RRID:CVCL_0027	
Cell line (*H. sapiens*)	Huh6	RCB	Cat#: RCB1367; RRID:CVCL_4381	
Cell line (*H. sapiens*)	Huh7	JCRB	Cat#: JCRB0403; RRID:CVCL_0336	
Cell line (*H. sapiens*)	MHCC97H	Zhongshan Hospital	RRID:CVCL_4972	Liver Cancer Institute of Zhongshan Hospital (Shanghai, China)
Cell line (*H. sapiens*)	PLC/PRF/5	ATCC	Cat#: CRL-802;RRID:CVCL_0485	
Cell line (*H. sapiens*)	SNU398	ATCC	Cat#: CRL-2233; RRID:CVCL_0077	
Cell line (*H. sapiens*)	SNU449	ATCC	Cat#: CRL-2234; RRID:CVCL_0454	
Cell line (*H. sapiens*)	SNU475	ATCC	Cat#: CRL-2236;RRID:CVCL_0497	
Cell line (*H. sapiens*)	SK-Hep1	ATCC	Cat#: HTB-52; RRID:CVCL_0525	
Cell line (*H. sapiens*)	LX2	ATCC	Cat#: SCC064;RRID:CVCL_5792	
Chemical compound, drug	Homoharringtonine	Selleck Chemicals	S9015	
Antibody	Anti-HSP90 (Mouse monoclonal)	Santa Cruz Biotechnology	Cat#: sc-13119;RRID:AB_675659	WB (1:5000)
Antibody	Anti-α-SMA (Mouse monoclonal)	Sigma-Aldrich	Cat#: A5228; RRID:AB_262054	WB (1:2000)IF (1:200)
Antibody	Anti-Collagen I (Rabbit polyclonal)	ProteinTech	Cat#: 14695-1-AP; RRID:AB_2082037	WB (1:2000)IF (1:200)
Sequence-based reagent	ACTA2_F	This paper	PCR primer	5′GACAATGGCTCTGGGCTCTGTAA3′
Sequence-based reagent	ACTA2_R	This paper	PCR primer	5′CTGTGCTTCGTCACCCACGTA3′
Sequence-based reagent	COL1A1_F	This paper	PCR primer	5′TCCTGGTCCTGCTGGCAAAGAA3′
Sequence-based reagent	COL1A1_R	This paper	PCR primer	5′CACGCTGTCCAGCAATACCTTGA3′
Software, algorithm	R software, version 3.6.0	https://cran.r-project.org/	RRID:SCR_001905	
Software, algorithm	ImageJ, version 1.53k	http://imagej.net/	RRID:SCR_003070	
Software, algorithm	Combenefit, version 2.02	https://sourceforge.net/projects/combenefit/		

### LINCS data source and processing

We downloaded the LINCS level 5 data (moderated Z-score) which comprises the differential expression signatures for nearly 20,000 unique compounds as well as meta-information of these signatures from Gene Expression Omnibus (GEO) database (Phase I: GSE92742, Phase II: GSE70138). Because this study only focused on analyzing compound signatures, those signatures induced by other perturbagens including gene knockdown (knockout) and gene overexpression were first excluded. The L1000 platform used by LINCS project only measures the expression level of 978 landmark genes, and the expression of remaining genes was based on imputation (Subramanian et al., 2017). This set of landmark genes is widely expressed in various cellular contexts and can well represent the full genome (Subramanian et al., 2017). Accordingly, we chose to use just the landmark genes (Chen et al., 2017a; Chen et al., 2017b). To ensure the reliability, only high-quality signatures are designated for following analyses (is_gold=1). In addition, L1000 data was further filtered for only 6-hr treatment samples due to the most abundant experiments on HepG2 cell line in this time point. Besides, the previous study also showed that gene expression changes obtained at a late time point (such as 24 hr) might reflect secondary or even tertiary responses, and the mechanistic effects of compounds might not be correctly recorded at late time point (De Wolf et al., 2016). As for the perturbation concentration, we selected expression profiles measured at 10 μM considering that this relatively high concentration is often chosen for performing high-throughput small molecular screens, and also, there exist the most abundant experiments at this concentration. The similarity between compound pairs was calculated based on cosine similarity algorithm (Cheng et al., 2013). For visualizing the LINCS data in 2D space, we measured the cosine distance (1−cosine similarity) between signatures and utilized cosine distance matrix as input to perform t-SNE analysis (Niepel et al., 2017). We downloaded the MOA and clinical phase information of compounds in LINCS from the Drug Repurposing Hub (https://clue.io/repurposing) (Corsello et al., 2017). The basal expression data of LINCS cell lines was achieved from the Cancer Cell Line Encyclopedia (CCLE) project (https://portals.broadinstitute.org/ccle/) (Ghandi et al., 2019). The expression-based similarity between different cell lines or between cell lines and clinical samples was determined through using ranked-based Spearman correlation (Chen et al., 2017b).

### AUC-based benchmarking standard

In this study, we mainly focused exclusively on one cell line (HepG2) and one disease (liver cancer). Two benchmarking standards, namely AUC-based standard and KS statistic-based standard, were generated for evaluating the retrieval performance of disease-compound similarity metrics across different conditions. For establishing AUC-based standard, we collected the drug response data from multiple data sources. Compound IC_50_s tested in HepG2 cell line was achieved from ChEMBL (version 27) (Mendez et al., 2019) and Liver Cancer Model Repository (LIMORE) (Qiu et al., 2019) data sets. Compounds among LINCS, ChEMBL, and LIMORE were mapped using compound name followed by manual inspection. Each experiment provided in ChEMBL was also manually checked to ensure the compliance with our requirement. Due to the redundancy of IC_50_s, the median IC_50_s of certain compounds among duplicates was used for representing the activity of this compound. We categorized the compounds into effective (IC_50_<10 μM) and ineffective groups (IC_50_≥10 μM) according to a previous study (Chen et al., 2017a). The ability to distinguish between effective and ineffective compounds was taken as a measurement of retrieval performance of different similarity metrics (namely AUC value) (Chen et al., 2017a). Notably, some have argued that partial AUC (limiting false positive rate at 0.1/0.01) might be a more relevant statistic for actual application of drug repositioning (Cheng et al., 2013; Cheng et al., 2014). However, due to the limited size of our benchmarking data set, adopting partial AUC could result in loss of statistical precision. The statistical significance of AUCs was calculated through performing permutation test. Briefly, we randomly permuted the class labels of the feature vectors and created 10,000 permutations to form a distribution of ‘random’ AUCs. Then, the p value was determined according to the fraction of ‘random’ AUCs greater than or equal to the observed AUC (Cheng et al., 2014). For distinguishing, AUC used for evaluating drug retrieval performance was renamed as drug retrieval-associated AUC (DR-AUC), the higher values of which indicate better performance.

### KS statistic-based benchmarking standard

To avoid confusion, it should be noted that KS-based method was also used for calculating disease-compound similarity scores, and the specific details were described below. For generating the benchmarking data set required for the KS statistic-based standard, we systematically surveyed clinical trials involved in HCC treatment and compiled a set of potential HCC agents (clinicaltrials.gov). Preliminary retrieval yielded 1999 results, and after removing trials failing to fulfill our requirements, we obtained 254 potential therapeutic agents for HCC. The detailed retrieval process was presented in Figure 3A. To minimize potential selection bias, this process was performed independently by two investigators (CY and XH). Perturbagen-induced expression profiles of 27 agents among these tested in HepG2 cell line are available in LINCS data set. Based on the assumption that HCC-associated agents are more likely to reverse HCC signature than random agent combinations, the enrichment capabilities of different similarity metrics could be used to assess their repositioning performance. The calculation of enrichment score (ES) of HCC agents was generally identical with that in gene set enrichment analysis (Subramanian et al., 2005). Considering that there was no need to account for the size of the agents set, we did not calculate the normalized enrichment score (NES) that might introduce additional randomization. To obtain a nominal p value, we created 10,000 permutations and recomputed the ES for each permutation to form a null distribution. The significance p of the observed ES was then determined relative to the null distribution. Notably, ES used in retrieval performance evaluation was renamed as drug retrieval-associated ES (DR-ES), the lower values of which represent better performance.

### Other pharmacogenomic data sets

In addition to ChEMBL and LIMORE, the drug response data of HepG2 cell line was also obtained from the Cancer Therapeutics Response Portal (CTRP) data set (CTRPv.2.0, released October 2015) (Rees et al., 2016). Considering that IC_50_s were not provided, the available AUDRC values were used solely for demonstrating the correlation between reversal potency and drug efficacy in different conditions. The AUC values in CTRP range from 0 to 30, and similar to IC_50_, lower values indicate increased sensitivity to treatment. To investigate the drug sensitivity of repositioning candidate across different HCC cell lines, we achieved the response data from the PRISM Repurposing data set (19Q4, released December 2019). Although IC_50_s are also provided by PRISM as one of drug response metrics, the drug response data of HepG2 cell line is absent (similar situation also exists in the Genomics of Drug Sensitivity in Cancer data set). Therefore, drug response data in these data sets were not used for developing AUC-based benchmarking standard.

### Genetic dependency data

CRISPR dependency data were obtained from the 20Q1 dependency map (DepMap) portal, which contained dependencies estimated for nearly 20,000 protein-coding genes and 739 cell lines using the CERES algorithm (Meyers et al., 2017). CERES score (gene effect) was used to measure the dependency of the gene of interest in cell lines, and a lower CERES score indicates a higher likelihood that the gene is essential in cell growth and survival. Besides, data of dependency probability were also achieved. A probability of dependency of certain gene in certain cell lines greater than 0.5 represents that the gene can be considered essential in this cell line. Essential genes in liver cancer were defined as genes that were essential in all 22 liver cancer cell lines.

### RNA-sequencing data sets

We collected five RNA-sequencing (RNA-seq)-based HCC cohorts, including CHCC-HBV (Gao et al., 2019), LICA-FR (Schulze et al., 2015), LIRI-JP (Fujimoto et al., 2016), TCGA-LIHC (Ally et al., 2017), and GSE124535 (Jiang et al., 2019), representing 947 HCC patients derived from four geographically different origins. Of these, LICA, LIRI, and LIHC cohorts provided raw counts quantifying gene expression, which were transformed into transcripts per kilobase million (TPM) values for subsequent analyses (raw counts were only used for *edgeR*-based differential expression analysis) (Li and Dewey, 2011). CHCC and GSE124535 cohorts provided fragments per kilobase per million reads (FPKM) normalized data, which was also converted to TPM values. All TPM values were log2 transformed. In addition, the batch effects-normalized expression matrices of ~10,000 patients across 33 human cancers (TCGA Pan-Cancer) were downloaded from the UCSC Xena browser (http://xena.ucsc.edu/). The RNA-seq data of 29 normal tissues were downloaded from the Genotype-Tissue Expression (GTEx) project (https://gtexportal.org/home/). Ensembl GeneIDs were mapped to HGNC symbols using *biomaRt* package.

### Array data sets

Five microarray-based clinical cohorts, including E-TABM-36 (Kim et al., 2012), GSE14520 (Roessler et al., 2012), GSE54236 (Villa et al., 2016), GSE76427 (Grinchuk et al., 2018), and GSE84005, were included to construct and validate HCC-associated signatures. Raw microarray data generated from Affymetrix platforms were normalized using robust multi-array average (RMA) method in *Affy* package (Gautier et al., 2004), while Illumina platform-derived raw data were normalized using the robust spline normalization (RSN) method in *lumi* package (Du et al., 2008). In other cases, normalized data were directly downloaded for use. Three liver cancer development-associated cohorts, including GSE89377, GSE6764 (Wurmbach et al., 2007), and GSE15654 (Hoshida et al., 2013), were also included for constructing the new signature, Sig_evo_, which was then applied to query LINCS for finding potential therapeutics of liver cancer. The samples in GSE89377 and GSE6764 covered multiple stages of the development of liver cancer, with the ability to recapitulate the step-wise process of hepatocarcinogenesis and progression. As for GSE15654, this cohort contains the gene expression profiles of samples from 216 patients with early cirrhosis who were prospectively followed for a median of 10 years, which thus can be used to identify the relationship between gene expression and HCC occurrence (Hoshida et al., 2013). In addition, a liver fibrosis-associated clinical cohort (GSE84044) (Wang et al., 2017) and four experimental data sets, including two carbon tetrachloride (CCl_4_)-treated mouse data sets (GSE27640 and GSE71379) (Fuchs et al., 2014; Nakagawa et al., 2016) and two diethylnitrosamine (DEN)-treated rat data sets (GSE19057 and GSE63726) (Fuchs et al., 2014; Nakagawa et al., 2016), were utilized to further assess the potential implication of Sig_evo_. Mouse and rat genes were mapped to orthologous human genes using *biomaRt* package, and genes without known human homologous relationships were excluded.

### Clinical data

Among the clinical cohorts above, nine cohorts have corresponding follow-up information, including four RNA-seq cohorts (CHCC, LICA, LIRI, and LIHC), and five microarray cohorts (E-TABM-36, GSE14520, GSE54236, GSE76427, and GSE15654). For RNA-seq cohorts, the survival data of CHCC and LICA cohort were obtained from the supplementary files of reference (Gao et al., 2019; Schulze et al., 2015), data of LIRI cohort were achieved from the International Cancer Genome Consortium (ICGC) portal (https://dcc.icgc.org/), and data of LIHC cohort were achieved from TCGA Pan-Cancer Clinical Data Resource (TCGA-CDR) (Liu et al., 2018). For microarray cohorts, complete clinical data were accessed from either public database (GEO: https://www.ncbi.nlm.nih.gov/gds/; ArrayExpress: https://www.ebi.ac.uk/arrayexpress/) or the original authors. Notably, except GSE15654 which uses the occurrence of HCC as endpoint, other eight cohorts all take survival status as endpoint.

### Signature matching methods

The retrieval performance of six different matching methods, including XSum (Cheng et al., 2014), XCos (Cheng et al., 2013; Cheng et al., 2014), XCor (Zhou et al., 2018), XSpe (Zhou et al., 2018), KS test (Lamb et al., 2006), and the RGES (Chen et al., 2017a), was systematically benchmarked. Based on the consideration that small variations in expression changes may be noise without biological significance, the eXtreme methods only utilized top up- and downregulated genes in compound signatures for similarity score calculation (all remaining genes were assigned the values of zero). By contrast, KS and RGES methods use complete compound profiles as reference signatures. The detailed features and scoring schemes of these methods are described as follows.

The XSum method handles the up- and downregulated genes separately. In brief, the sums of the change values in reference/compound signatures relative to upregulated query/disease genes (sum_up_) and downregulated query/disease genes (sum_down_) are first calculated. Then, XSum is defined as following: XSum=sum_up_−sum_down_. Other three eXtreme methods, including XCos, XCor, and XSpe, take disease signatures as a whole to query compound signatures, and they calculated the correlation between the numeric vectors of disease and compound signatures using cosine similarity, Pearson correlation, and spearman correlation, respectively. Notably, cosine similarity is nearly identical with Pearson correlation except without centering vectors around the mean values. The KS method was adopted by the first CMap study and has been the most widely used method for connecting disease signatures to compound signatures (Lamb et al., 2006). Similar to XSum, KS method also need to seperate disease signatures into two gene sets, upregulated gene set and downregulated gene set, and ignores the magnitude of differential expression. Briefly, using complete compound profiles as reference, maximum deviation (MD)-based ES of upregulated gene set (es_up_) and downregulated gene set (es_down_) are first computed. If es_up_ and es_down_ have the same algebraic sign then KSscore=0, otherwise, KSscore=es_up_−es_down_. The RGES method is a recently proposed modification of the original KS method, which was demonstrated to perform better in drug response prediction than KS method (Chen et al., 2017a). In contrast to original KS method, RGES focuses on the reversal relation between the disease and agents, and RGES is defined as es_up_−es_down_ regardless of the sign of es_up_ and es_down_. In addition to the above six methods, there also exist many other methods, such as WSS/sscMap (Zhang and Gant, 2008), TES (Iorio et al., 2010), ProbCmap (Parkkinen and Kaski, 2014), NFFinder (Setoain et al., 2015), and EMUDRA (Zhou et al., 2018), for calculating the similarity between disease and compound signatures. However, some of them are not accessible currently and some are developed based on the data of initial CMap data set (1309 compounds). Accordingly, we did not include these methods in our analyses.

### Generation of query signatures for performance evaluation

For evaluating retrieval performance of similarity metrics at different conditions, we prepared four HCC-associated gene signatures to query LINCS. Two of them, Sig_gastro_ and Sig_NC_, are achieved from previously published studies (Chen et al., 2017a; Chen et al., 2017b). Given that the development of these two signatures was mainly based on LIHC cohort, as a complement, the other two were generated from another RNA-seq cohort (LIRI) and a microarray cohort (GSE54236), respectively. The differentially expressed genes in LIRI cohort were computed using *edgeR* package (version 3.26.5) on raw count data (McCarthy et al., 2012). For microarray data, we used *limma* package (version 3.40.2) to conduct differential expression analysis on normalized data (Ritchie et al., 2015). The statistically significant differential genes in the above analyses were defined as adjusted p<0.01 and absolute log_2_ fold change (FC)>1. As a result, we obtained a 70-gene signature (Sig_LIRI_) with 48 up- and 22 downregulated genes from LIRI and a 28-gene signature (Sig_GSE54236_) with 22 up- and 6 downregulated genes from GSE54236, respectively, which could represent discordant expression pattern of HCC. The gene numbers in signatures created through differential expression analysis were much less than that in prognostic signatures (see section below). To make these two types of signatures comparable, we relaxed the significance threshold of differential genes to p<0.01 and log_2_FC>0.5, and built two increased signatures which included 125 (LIRI) and 116 genes (GSE54236). These two increased signatures were also used to explore the potential influences of signature size.

### Construction of size-diversified query signatures

We adopted two independent approaches to explore whether the differences of query/disease signature size could affect subsequent drug retrieval. The first approach was based on iterating the threshold of fold change values, ranging from 0.1 to 0.1 to the maximum/minimum with an increment/decrement of 0.05, which could obtain a number of query signatures with varying signature size (duplicates were removed). As for the second approach, two increased signatures, 125-gene signature from LIRI and 116-gene signature from GSE54236, were taken as the basis for generating smaller-size testing signatures. Briefly, we randomly extracted testing signatures from complete signatures, with the size ranging from the minimum of 5 to the maximum of 124 or 115. To avoid bias, this process was repeated 1000 times to generate 1000 testing signatures for each signature size.

### Construction of query signatures representing different clinical phenotypes

To investigate whether the clinical phenotype of signature was potential factor affecting the retrieval performance, we developed two strategies, a forward strategy starting from generation of signatures with distinguishing clinical phenotypes to the evaluation of retrieval performance and a backward strategy starting from obtaining signature with the best performance to the comprehensive investigations of its clinical implication. For the first strategy, to compare with general HCC signature representing discordant expression pattern, two prognostic signatures based on LIRI and GSE54236 cohorts were constructed. We integrated survival data with expression data and performed Cox proportional hazards regression to assess association between overall survival and gene expression. The statistically significant prognostic genes were defined as p<0.005. A 133-gene prognostic signature with 117 poor- and 16 good-outcome genes was generated based on LIRI, while analysis on GSE54236 resulted in a 107-gene prognostic signature with 79 poor- and 28 good-outcome genes. Comparisons of drug retrieval performance between these two types of signatures were carried out subsequently. For the second strategy, taking 978 landmark genes as a basis, simple random sampling without replacement (SRSWOR) was performed to extract genes from landmarks for forming candidate signatures. The size of randomized signatures was set at 100 and the process of random sampling was repeated 10,000 times to obtain a collection of 10,000 randomized signatures. The DR-AUC and DR-ES values were then calculated for each signature, and the optimal one was defined as the signature with the minimum of DR-AUC multiplying DR-ES.

### Generation of evolution-associated query signature

To find compounds with potential to prevent and treat liver cancer, we developed a hepatocarcinogenesis and progression-associated signature. GSE89377 cohort was utilized to build this signature while GSE6764 cohort was taken for external validation. To identify genes associated with developmental stages, RF model was constructed, taking stages as dependent variable. Variable importance was assessed with the mean decrease accuracy (MDA) measures for individual factors in RF model. Variables with positive MDA values are of high importance in predicting stages. In other words, these variables are more likely to be related with liver cancer development and progression (negative MDA values can be regarded as equivalent to zero importance with no predictive power). RF analysis was independently repeated 1000 times with 1000 trees growing each time, and genes with positive MDA values incorporated in more than 200 iterations were kept for subsequent analyses.

We next performed WGCNA to assign resultant genes into modules according to expression similarity and recognize the trajectories of gene expression during liver cancer development (Langfelder and Horvath, 2008). First, an appropriate soft threshold was estimated by using the *pickSoftThreshold* function in *WGCNA* package. Then, we constructed WGCNA network and detected gene expression modules using *blockwiseModules* function with a minimum module gene number of 50, soft thresholded power of 12, and a dendrogram cut height of 0.3. Genes without assignment to specific modules were assigned the color of gray. Module eigengenes (MEs) representing the first principal components (PC1) of each module were returned, and the module-trait relationship (MTR) analysis was conducted by calculating the correlation between MEs and developmental stages. The expression trend of each module across seven stages of HCC development was visualized through using mean PC1 values of samples in each stage to generate trend curves. According to the correlation coefficient of MTR analysis and the visualized expression trend of each module, two modules exhibiting the highest positive/negative correlation with developmental stages as well as showing gradually increasing or decreasing expression trends were selected. Subsequently, to explore the biological processes associated with genes in these two modules, we conducted hypergeometric test based on the hallmark gene sets (h.all.v7.0.symbols) downloaded from the Molecular Signatures Database (MSigDB) using *enricher* function in *clusterProfiler* package (Liberzon et al., 2015; Yu et al., 2012). The p values from the hypergeometric tests were adjusted for multiple comparison testing and an adjusted p value less than 0.05 was considered significantly enriched.

Genes in these two modules were mapped to the 978 landmark genes, resulting in a 159-gene panel (134 genes in ascending module and 25 genes in descending module). According to the findings described in Results section, we further narrowed down this panel to create a query signature with 100 genes. The molecular and clinical data in GSE15654 were utilized to determine the association between the expression patterns of signatures and the occurrence of HCC. Briefly, we first performed SRSWOR to extract a subset of 100 genes from the 159-gene panel, repeated 10,000 times. As a result, 10,000 randomized signatures with 100 genes per signature were generated. Next, PC1 values of all randomized signatures were extracted based on expression data from GSE15654 to represent the overall expression patterns of these signatures, and the follow-up data using HCC occurrence as endpoint was then integrated with above expression pattern data for subsequent Cox proportional hazards regression (COXPH). The signature which had the minimum p value across the 10,000 COXPH analyses was considered the optimal signature. The expression trend of this signature was further validated GSE6764 cohort.

### Human cell lines and compounds

The liver cancer cell lines, Hep3B, Huh7, PLC/PRF/5, SNU398, and Huh6, were provided by Erasmus University (Rotterdam, the Netherlands). MHCC97H and SK-Hep1 were provided by the Liver Cancer Institute of Zhongshan Hospital (Shanghai, China). SNU449, SNU475, HepG2, and the immortalized human HSC line LX2 were purchased from the American Type Culture Collection (ATCC). These cells were maintained in Dulbecco’s modified Eagle’s medium (DMEM) (Gibco, Carlsbad, CA) supplemented with 10% fetal bovine serum (FBS) (Gibco) and 1% penicillin/streptomycin (Basal Media), incubated at 37°C in humidified atmosphere with 5% CO2. Mycoplasma contamination was excluded via a PCR-based method. The identities of all the cell lines were confirmed by short tandem repeat (STR) profiling. Human recombinant transforming growth factor β1 (TGF-β1) was purchased from R&D Systems (Minneapolis, MN), which was used to activate LX2 (10 ng/ml TGF-β1 for 24 hr). HHT treatment was performed by pre-treating for 2 hr before TGF-β1 stimulation. HHT (S9015) was purchased from Selleck Chemicals and dissolved in dimethyl sulfoxide (DMSO) using a storage concentration of 10 mM.

### Cell proliferation assays

For long-term cell proliferation assay, cells were seeded into six-well plates (2–3×10^4^ cells per well) and HHT was added after 24 hr. Cells were treated with HHT as indicated for 10 days during which the culture media were replaced every 3 days. Then, cells were stained with 1% crystal violet for 10 min and rinsed with tap water. Pictures were taken using ImageScanner III (GE Healthcare) at 300-dpi resolution. For IncuCyte real-time assay, cells were cultured and seeded into 96-well plates at a density of 1000–1500 cells per well, and 24 hr later, HHT was added at indicated concentrations. Cells were imaged every 4 hr in IncuCyte ZOOM system (Essen Bioscience) and phase-contrast images were collected and analyzed to determine the proliferation curves based on cell confluence. Cell viability in dose-response matrix was assessed using CellTiter-Blue (CTB) assay (Promega) according to the manufacturer’s recommendations. For measuring the synergistic effect of HHT-sorafenib combination, three different models, including Bliss independence model, Loewe additivity model and Highest single agent (HAS) model, were adopted, which were all implemented in Combenefit software version 2.02 (Di Veroli et al., 2016).

### Xenografts

Male BALB/c nude mice of 6–8 weeks old were used to establish xenograft tumor model. MHCC97H cells were suspended in 200-µl serum-free DMEM and subcutaneously injected into the upper flank of each mouse. When tumors reached a volume of approximately 50–100 mm^3^, mice from both groups were randomly assigned to treatment with vehicle or HHT (1 mg/kg, daily gavage). The 1 mg/kg dosage of HHT used to treat the nude mice with xenograft tumors was selected according to previous studies (Wang et al., 2021; Weng et al., 2018). Tumor volume was monitored every 3–4 days. The body weights were monitored every day. After 2 weeks of treatment, the mice were euthanized, the tumors were weighed and imaged.

### Liver fibrosis model

Mouse model of liver fibrosis was established based on previous publications (Chen et al., 2014; Qu et al., 2018; Scholten et al., 2015). In specific, 6-week-old male C57BL/6 mice (Shanghai Model Organisms Center, Shanghai, China) were treated three times a week for 10 weeks with intragastric administration of 0.1 ml of a 40% solution of CCl_4_ (Aladdin, Shanghai, China) in olive oil (N=12) or olive oil alone (N=6). A subset of CCL4-treated mice received daily gavage of either 0.2 mg/kg HHT (N=6) or vehicle (N=6) during weeks 6–10. A concentration of 0.2 mg/kg was selected considering that the concentration for prevention is typically much lower than that for anti-tumor therapy (Bayo et al., 2021). Mice were sacrificed 3 days after the final treatment. The liver was harvested and cardiac terminal blood draw was also performed.

### Histology

Formalin-fixed samples were embedded in paraffin, cut into 5-µm-thick sections. Histologic slides were stained with hematoxylin and eosin (H&E), Masson’s trichrome, and Sirius Red according to standard procedures, and then scanned using the Aperio CS Scanscope (Aperio Technologies, CA, USA). Fibrosis score was assessed on Masson’s trichrome staining using Ishak scoring system (Ishak, 1994) and the positive area of Sirius Red staining was quantified by ImageJ software (version 1.53k, http://imagej.net/). All slides were reviewed in a blinded fashion by the same expert pathologist.

### Liver function tests

Blood was collected by a cardiac blood draw at the time of sacrifice. Blood was allowed to clot at least 20 min and serum was purified by centrifugation. Serum was stored at –80℃ prior to use. Liver function was evaluated through measuring several serological markers, including ALT, AST, alkaline phosphatase (ALP), and albumin (Alb). The serum levels of these markers were determined by BS-200 Chemistry Analyzer (Mindray, China).

### Quantitative real-time PCR

We first harvested cells using TRIzol reagent (Invitrogen) based on the manufacturer’s instruction. Then, cDNA synthesis was carried out using Maxima Universal First Strand cDNA Synthesis Kit (No. K1661, Thermo Fisher Scientific). Quantitative reverse transcription PCR (qRT-PCR) assays were conducted using 7500 Fast Real-Time PCR System (Applied Biosystems). Relative mRNA levels of genes shown were normalized to the mRNA level of glyceraldehyde-3-phosphate dehydrogenase (GAPDH) (housekeeping gene). The primer sequences for assays using SYBR Green master mix (Roche) are as follows:

β-actin Forward, 5′AAATCTGGCACCACACCTTC3′,
β-actin Reverse, 5′GGGGTGTTGAAGGTCTCAAA3′,
Collagen I Forward, 5′TCCTGGTCCTGCTGGCAAAGAA3′,
Collagen I Reverse, 5′CACGCTGTCCAGCAATACCTTGA3′,
α-SMA Forward, 5′GACAATGGCTCTGGGCTCTGTAA3′,
α-SMA Reverse, 5′CTGTGCTTCGTCACCCACGTA3′.

### Western blotting analysis

Cells were washed with PBS and lysed on ice with RIPA lysis buffer supplemented with Complete Protease Inhibitor (Roche) and Phosphatase Inhibitor Cocktails II and III (Sigma). Protein concentration was measured using the BCA Protein Assay Kit (Pierce). All lysates were then freshly prepared and processed with Novex NuPAGE Gel Electrophoresis Systems (Thermo Fisher Scientific) followed by western blotting. The antibody against α-smooth muscle actin (α-SMA) (A5228) was obtained from Sigma-Aldrich (USA) and the antibody against collagen I (14695-1-AP) was achieved from ProteinTech.

### Immunofluorescence

Cells were cultured on glass cover slips, fixed for 10 min with 4% formaldehyde, and permeabilized with 0.5% Triton X-100 for 15 min at room temperature. Immunofluorescence analysis was performed using the following antibodies: anti-Actin, α-Smooth Muscle antibody (1:200), anti-collagen I (1:200), anti-mouse IgG Fab2 Alexa Fluor (R) 488 (1:2000, CST), and anti-rabbit IgG Fab2 Alexa Fluor (R) 542 (1:2000, CST). Cell nuclei were stained with DAPI (4,6-diamidino-2-phenylindole). After immunostaining, the samples were observed using a LEICA TCS SP5 confocal microscope.

### RNA sequencing

For RNA sequencing, total RNA was extracted and purified using the TRIzol reagent (Invitrogen). The library was prepared using TruSeq RNA sample prep kit according to the manufacturer’s protocol (Illumina). Paired-end libraries were sequenced by an Illumina HiSeq 4000, with a sequence coverage of 20 million paired reads. For data analysis, raw sequencing reads were mapped to the human genome (GRCh38) using STAR (version 2.4.2g1) (Dobin et al., 2013). Then gene-level read counts were generated using featureCounts from the subRead package with default settings (Liao et al., 2014).

### Statistics

All the computational analyses and graphical visualization were performed in R software, version 3.6.0 (https://cran.r-project.org/). Unless stated otherwise, correlation between two continuous variables was measured by Spearman’s rank-order correlation, and pairwise comparisons were conducted using Kruskal-Wallis and Wilcoxon sum-rank tests. ROC curves and AUC values were visualized and calculated using the pROC package (Robin et al., 2011). The hazard ratio was estimated using Cox regression model in *survival* R package. Cumulative hazard curve was carried out using *jskm* package and the log-rank test was used to determine the statistical significance of differences. All data points indicate individual biologic replicates (independent experimental samples) and not technical replicates (the same sample re-analyzed using the same method). A two-tailed p<0.05 was considered significant unless indicated otherwise.

## Data Availability

Sequencing data have been deposited in GEO under accession codes GSE180243 and GSE193897. All data generated or analysed during this study are included in the manuscript and supporting files. The following datasets were generated: ChenY 2021A survey of optimal strategy for signature-based drug repositioning and an application to liver cancerNCBI Gene Expression OmnibusGSE18024310.7554/eLife.71880PMC889372135191375 ChenY 2022A survey of optimal strategy for signature-based drug repositioning and an application to liver cancer (liver cancer cell lines)NCBI Gene Expression OmnibusGSE19389710.7554/eLife.71880PMC889372135191375 The following previously published datasets were used: Aurélien deR CharlesB DavidR DominiqueF EmmanuelleJ JacquesB JeanS JessicaZ-R PauletteB-S PierreL-P SandraR SandrineB 2007Transcription profiling of 57 hepato cellular carcinoma tumoral samples, 3 hepatocellular adenomas, 5 non-tumoral poolsArrayExpressE-TABM-36 JiangY ZhangL 2019Gene expression profiles of 35 paired HCC and non-tumor tissues by RNA-seq dataNCBI Gene Expression OmnibusGSE124535 AllyA BalasundaramM CarlsenR ChuahE ClarkeA DhallaN 2016Comprehensive and integrative genomic characterization of hepatocellular carcinomaXena Functional Genomics ExplorerTCGA Liver Cancer (LIHC)10.1016/j.cell.2017.05.046PMC568077828622513 GhandiM HuangFW Jané-ValbuenaJ KryukovGV LoCC McDonaldER 2018Broad Institute Cancer Cell Line Encyclopedia (CCLE)DepMap PortalCCLE GTEx Consortium 2017The Genotype-Tissue Expression (GTEx) projectGenotype-Tissue Expression (GTEx)GTEx Cancer Genome Atlas Research Network 2016The Cancer Genome Atlas Pan-Cancer analysis projectXena Functional Genomics ExplorerTCGA Pan-Cancer (PANCAN) SubramanianA NarayanR CorselloSM PeckDD NatoliTE LuX 2017A Next Generation Connectivity Map: L1000 Platform and the First 1,000,000 ProfilesCMap LINCS Gene Expression ResourceLINCS10.1016/j.cell.2017.10.049PMC599002329195078 MendezD GaultonA BentoAP ChambersJ De VeijM FélixE 2019ChEMBL: towards direct deposition of bioassay dataEuropean Bioinformatics InstituteChEMBL10.1093/nar/gky1075PMC632392730398643 ReesMG Seashore-LudlowB CheahJH AdamsDJ PriceEV GillS 2016Cancer Therapeutics Response PortalBroad InstituteCTRP QiuZ LiH ZhangZ ZhuZ HeS WangX 2019Liver Cancer Model RepositoryLIMORELIMORE CorselloSM NagariRT SpanglerRD RossenJ KocakM BryanJG 2020PRISM Repurposing datasetDepMap PortalPRISM WangXW 2010Gene expression data of human hepatocellular carcinoma (HCC)NCBI Gene Expression OmnibusGSE14520 VillaE CritelliR LeiB MarzocchiG CammàC GiannelliG PontissoP ColopiS CaporaliC CabibboG MilosaF MaccioL Martinez-ChantarML TodescaP TurolaE BerselliA De MariaN BallestriS SchepisF LoriaP GerundaGE LosiL Di BenedettoF CilloU 2014Prospective gene expression analysis of human RNA samples from Hepatocellular Carcinoma in relation with growth rate and survivalNCBI Gene Expression OmnibusGSE54236 GrinchukOV YenamandraSP KuznetsovVA 2017Microarray expression data for tumor and adjacent non-tumor tissues from hepatocellular carcinoma patientsNCBI Gene Expression OmnibusGSE76427 TuX SongJ ChenX HeF ZhouG 2017Integrative omics analysis in HCC samples [mRNA expression]NCBI Gene Expression OmnibusGSE84005 WurmbachE ChenY KhitrovG ZhangW RoayaieS SchwartzM FielI ThungS MazzaferroV BruixJ BottingerE FriedmanS WaxmanS LlovetJM 2007Genome-wide molecular profiles of HCV-induced dysplasia and hepatocellular carcinomaNCBI Gene Expression OmnibusGSE676410.1002/hep.2162217393520 HoshidaY VillanuevaA SangiovanniA SoleM GouldJ GuptaS TaylorB CrenshawA GabrielS MinguezB IavaroneM FriedmanS ColomboM LlovetJM GolubTR 2013Gene-expression profiles of hepatitis C-related, early-stage liver cirrhosisNCBI Gene Expression OmnibusGSE15654 EunJ NamS 2017Identifying novel drivers of human hepatocellular carcinoma and revealing clinical relevance as early diagnostic and prognostic biomarkerNCBI Gene Expression OmnibusGSE89377 WangM LuL ZhangJ YuanZ ZhangX 2016Characterization of gene expression profile in HBV-related liver fibrosis patientsNCBI Gene Expression OmnibusGSE8404410.1038/srep43446PMC533797828262670 FuchsBC HoshidaY FujiiT YamadaS LauwersGY McGinnCM WeiL KurodaT LanutiM GuptaS CrenshawA OnofrioR TaylorB WincklerW GolubTR TanabeKK 2014Gene expression profile of liver tissue in carbon tetrachloride (CCl4)-treated mouse treated with erlotinibNCBI Gene Expression OmnibusGSE27640 FuchsBC HoshidaY 2016Gene expression profile of liver tissue from carbon tetrachloride (CCl4)-treated mouse cultured ex vivoNCBI Gene Expression OmnibusGSE71379 FuchsBC HoshidaY FujiiT LauwersGY McGinnCM YamadaS KurodaT LanutiM GolubTR TanabeKK 2014Gene expression profile of liver tissue in low-dose, repeated diethylnitrosamine (DEN)-treated rat treated with erlotinibNCBI Gene Expression OmnibusGSE19057 FuchsBC HoshidaY 2016Gene expression profiles of fractionated cells from cirrhotic rat liversNCBI Gene Expression OmnibusGSE63726

## Additional files

Supplementary file 1. Pharmacogenomic and transcriptomic datasets used in this study.

Supplementary file 2. Cosine similarity within different drugs of LINCS.

Supplementary file 3. Benchmarking dataset for AUC-based standard.

Supplementary file 4. Benchmarking dataset for KS statistic-based standard.

Supplementary file 5. A summary of four query signatures used for benchmarking signature matching methods.

Supplementary file 6. Results of AUC/KS-based performance evaluation of six signature matching methods.

Supplementary file 7. The list of genes in Sig(evo).

Supplementary file 8. Results of computational drug repositioning for liver cancer.

Transparent reporting form

## References

[bib1] Abou-Alfa GK, Meyer T, Cheng A-L, El-Khoueiry AB, Rimassa L, Ryoo B-Y, Cicin I, Merle P, Chen Y, Park J-W, Blanc J-F, Bolondi L, Klümpen H-J, Chan SL, Zagonel V, Pressiani T, Ryu M-H, Venook AP, Hessel C, Borgman-Hagey AE, Schwab G, Kelley RK (2018). Cabozantinib in Patients with Advanced and Progressing Hepatocellular Carcinoma. The New England Journal of Medicine.

[bib2] Ally A, Balasundaram M, Carlsen R, Chuah E, Clarke A, Dhalla N, Ma YJC (2017). Comprehensive and Integrative Genomic Characterization of Hepatocellular Carcinoma. Cell.

[bib3] Ashburn TT, Thor KB (2004). Drug repositioning: identifying and developing new uses for existing drugs. Nature Reviews. Drug Discovery.

[bib4] Bayo J, Fiore EJ, Dominguez LM, Cantero MJ, Ciarlantini MS, Malvicini M, Atorrasagasti C, Garcia MG, Rossi M, Cavasotto C, Martinez E, Comin J, Mazzolini GD (2021). Bioinformatic analysis of RHO family of GTPases identifies RAC1 pharmacological inhibition as a new therapeutic strategy for hepatocellular carcinoma. Gut.

[bib5] Bray F, Ferlay J, Soerjomataram I, Siegel RL, Torre LA, Jemal A (2018). Global cancer statistics 2018: GLOBOCAN estimates of incidence and mortality worldwide for 36 cancers in 185 countries. CA.

[bib6] Bruix J, Qin S, Merle P, Granito A, Huang Y-H, Bodoky G, Pracht M, Yokosuka O, Rosmorduc O, Breder V, Gerolami R, Masi G, Ross PJ, Song T, Bronowicki J-P, Ollivier-Hourmand I, Kudo M, Cheng A-L, Llovet JM, Finn RS, LeBerre M-A, Baumhauer A, Meinhardt G, Han G, RESORCE Investigators (2017). Regorafenib for patients with hepatocellular carcinoma who progressed on sorafenib treatment (RESORCE): a randomised, double-blind, placebo-controlled, phase 3 trial. Lancet (London, England).

[bib7] Chan J, Wang X, Turner JA, Baldwin NE, Gu J (2019). Breaking the paradigm: Dr Insight empowers signature-free, enhanced drug repurposing. Bioinformatics (Oxford, England).

[bib8] Chen X, Gan Y, Li W, Su J, Zhang Y, Huang Y, Roberts AI, Han Y, Li J, Wang Y, Shi Y (2014). The interaction between mesenchymal stem cells and steroids during inflammation. Cell Death & Disease.

[bib9] Chen B, Ma L, Paik H, Sirota M, Wei W, Chua MS, So S, Butte AJ (2017a). Reversal of cancer gene expression correlates with drug efficacy and reveals therapeutic targets. Nature Communications.

[bib10] Chen B, Wei W, Ma L, Yang B, Gill RM, Chua MS, Butte AJ, So S (2017b). Computational Discovery of Niclosamide Ethanolamine, a Repurposed Drug Candidate That Reduces Growth of Hepatocellular Carcinoma Cells In Vitro and in Mice by Inhibiting Cell Division Cycle 37 Signaling. Gastroenterology.

[bib11] Cheng J, Xie Q, Kumar V, Hurle M, Freudenberg JM, Yang L, Agarwal P (2013). Evaluation of analytical methods for connectivity map data. Pacific Symposium on Biocomputing. Pacific Symposium on Biocomputing.

[bib12] Cheng J, Yang L, Kumar V, Agarwal P (2014). Systematic evaluation of connectivity map for disease indications. Genome Medicine.

[bib13] Corsello SM, Bittker JA, Liu Z, Gould J, McCarren P, Hirschman JE, Johnston SE, Vrcic A, Wong B, Khan M, Asiedu J, Narayan R, Mader CC, Subramanian A, Golub TR (2017). The Drug Repurposing Hub: a next-generation drug library and information resource. Nature Medicine.

[bib14] Corsello SM, Nagari RT, Spangler RD, Rossen J, Kocak M, Bryan JG, Humeidi R, Peck D, Wu X, Tang AA, Wang VM, Bender SA, Lemire E, Narayan R, Montgomery P, Ben-David U, Garvie CW, Chen Y, Rees MG, Lyons NJ, McFarland JM, Wong BT, Wang L, Dumont N, O’Hearn PJ, Stefan E, Doench JG, Harrington CN, Greulich H, Meyerson M, Vazquez F, Subramanian A, Roth JA, Bittker JA, Boehm JS, Mader CC, Tsherniak A, Golub TR (2020). Discovering the anti-cancer potential of non-oncology drugs by systematic viability profiling. Nature Cancer.

[bib15] De Wolf H, De Bondt A, Turner H, Göhlmann HWH (2016). Transcriptional Characterization of Compounds: Lessons Learned from the Public LINCS Data. Assay and Drug Development Technologies.

[bib16] Di Veroli GY, Fornari C, Wang D, Mollard S, Bramhall JL, Richards FM, Jodrell DI (2016). Combenefit: an interactive platform for the analysis and visualization of drug combinations. Bioinformatics (Oxford, England).

[bib17] Dobin A, Davis CA, Schlesinger F, Drenkow J, Zaleski C, Jha S, Batut P, Chaisson M, Gingeras TR (2013). STAR: ultrafast universal RNA-seq aligner. Bioinformatics (Oxford, England).

[bib18] Du P, Kibbe WA, Lin SM (2008). lumi: a pipeline for processing Illumina microarray. Bioinformatics (Oxford, England).

[bib19] Dudley JT, Sirota M, Shenoy M, Pai RK, Roedder S, Chiang AP, Morgan AA, Sarwal MM, Pasricha PJ, Butte AJ (2011). Computational repositioning of the anticonvulsant topiramate for inflammatory bowel disease. Science Translational Medicine.

[bib20] El-Khoueiry AB, Sangro B, Yau T, Crocenzi TS, Kudo M, Hsu C, Kim TY, Choo SP, Trojan J, Welling TH, Meyer T, Kang YK, Yeo W, Chopra A, Anderson J, Dela Cruz C, Lang L, Neely J, Tang H, Dastani HB, Melero I (2017). Nivolumab in patients with advanced hepatocellular carcinoma (CheckMate 040): an open-label, non-comparative, phase 1/2 dose escalation and expansion trial. Lancet (London, England).

[bib21] Finn RS, Qin S, Ikeda M, Galle PR, Ducreux M, Kim TY, Kudo M, Breder V, Merle P, Kaseb AO, Li D, Verret W, Xu DZ, Hernandez S, Liu J, Huang C, Mulla S, Wang Y, Lim HY, Zhu AX, Cheng AL, IMbrave150 Investigators (2020a). Atezolizumab plus Bevacizumab in Unresectable Hepatocellular Carcinoma. The New England Journal of Medicine.

[bib22] Finn RS, Ryoo BY, Merle P, Kudo M, Bouattour M, Lim HY, Breder V, Edeline J, Chao Y, Ogasawara S, Yau T, Garrido M, Chan SL, Knox J, Daniele B, Ebbinghaus SW, Chen E, Siegel AB, Zhu AX, Cheng AL, KEYNOTE-240 investigators (2020b). Pembrolizumab As Second-Line Therapy in Patients With Advanced Hepatocellular Carcinoma in KEYNOTE-240: A Randomized, Double-Blind, Phase III Trial. Journal of Clinical Oncology.

[bib23] Fuchs BC, Hoshida Y, Fujii T, Wei L, Yamada S, Lauwers GY, McGinn CM, DePeralta DK, Chen X, Kuroda T, Lanuti M, Schmitt AD, Gupta S, Crenshaw A, Onofrio R, Taylor B, Winckler W, Bardeesy N, Caravan P, Golub TR, Tanabe KK (2014). Epidermal growth factor receptor inhibition attenuates liver fibrosis and development of hepatocellular carcinoma. Hepatology (Baltimore, Md.).

[bib24] Fujimoto A, Furuta M, Totoki Y, Tsunoda T, Kato M, Shiraishi Y, Tanaka H, Taniguchi H, Kawakami Y, Ueno M, Gotoh K, Ariizumi S-I, Wardell CP, Hayami S, Nakamura T, Aikata H, Arihiro K, Boroevich KA, Abe T, Nakano K, Maejima K, Sasaki-Oku A, Ohsawa A, Shibuya T, Nakamura H, Hama N, Hosoda F, Arai Y, Ohashi S, Urushidate T, Nagae G, Yamamoto S, Ueda H, Tatsuno K, Ojima H, Hiraoka N, Okusaka T, Kubo M, Marubashi S, Yamada T, Hirano S, Yamamoto M, Ohdan H, Shimada K, Ishikawa O, Yamaue H, Chayama K, Miyano S, Aburatani H, Shibata T, Nakagawa H (2016). Whole-genome mutational landscape and characterization of noncoding and structural mutations in liver cancer. Nature Genetics.

[bib25] Fujiwara N, Friedman SL, Goossens N, Hoshida Y (2018). Risk factors and prevention of hepatocellular carcinoma in the era of precision medicine. Journal of Hepatology.

[bib26] Gao Q, Zhu H, Dong L, Shi W, Chen R, Song Z, Huang C, Li J, Dong X, Zhou Y, Liu Q, Ma L, Wang X, Zhou J, Liu Y, Boja E, Robles AI, Ma W, Wang P, Li Y, Ding L, Wen B, Zhang B, Rodriguez H, Gao D, Zhou H, Fan J (2019). Integrated Proteogenomic Characterization of HBV-Related Hepatocellular Carcinoma. Cell.

[bib27] Gautier L, Cope L, Bolstad BM, Irizarry RA (2004). affy--analysis of Affymetrix GeneChip data at the probe level. Bioinformatics (Oxford, England).

[bib28] Ghandi M, Huang FW, Jané-Valbuena J, Kryukov GV, Lo CC, McDonald ER, Barretina J, Gelfand ET, Bielski CM, Li H, Hu K, Andreev-Drakhlin AY, Kim J, Hess JM, Haas BJ, Aguet F, Weir BA, Rothberg MV, Paolella BR, Lawrence MS, Akbani R, Lu Y, Tiv HL, Gokhale PC, de Weck A, Mansour AA, Oh C, Shih J, Hadi K, Rosen Y, Bistline J, Venkatesan K, Reddy A, Sonkin D, Liu M, Lehar J, Korn JM, Porter DA, Jones MD, Golji J, Caponigro G, Taylor JE, Dunning CM, Creech AL, Warren AC, McFarland JM, Zamanighomi M, Kauffmann A, Stransky N, Imielinski M, Maruvka YE, Cherniack AD, Tsherniak A, Vazquez F, Jaffe JD, Lane AA, Weinstock DM, Johannessen CM, Morrissey MP, Stegmeier F, Schlegel R, Hahn WC, Getz G, Mills GB, Boehm JS, Golub TR, Garraway LA, Sellers WR (2019). Next-generation characterization of the Cancer Cell Line Encyclopedia. Nature.

[bib29] Grinchuk OV, Yenamandra SP, Iyer R, Singh M, Lee HK, Lim KH, Chow PK-H, Kuznetsov VA (2018). Tumor-adjacent tissue co-expression profile analysis reveals pro-oncogenic ribosomal gene signature for prognosis of resectable hepatocellular carcinoma. Molecular Oncology.

[bib30] Hoshida Y, Fuchs BC, Tanabe KK (2012). Prevention of hepatocellular carcinoma: potential targets, experimental models, and clinical challenges. Current Cancer Drug Targets.

[bib31] Hoshida Y, Villanueva A, Sangiovanni A, Sole M, Hur C, Andersson KL, Chung RT, Gould J, Kojima K, Gupta S, Taylor B, Crenshaw A, Gabriel S, Minguez B, Iavarone M, Friedman SL, Colombo M, Llovet JM, Golub TR (2013). Prognostic gene expression signature for patients with hepatitis C-related early-stage cirrhosis. Gastroenterology.

[bib32] Iorio F, Bosotti R, Scacheri E, Belcastro V, Mithbaokar P, Ferriero R, Murino L, Tagliaferri R, Brunetti-Pierri N, Isacchi A, di Bernardo D (2010). Discovery of drug mode of action and drug repositioning from transcriptional responses. PNAS.

[bib33] Ishak KG (1994). Chronic hepatitis: morphology and nomenclature. Modern Pathology.

[bib34] Jiang Y, Sun A, Zhao Y, Ying W, Sun H, Yang X, Xing B, Sun W, Ren L, Hu B, Li C, Zhang L, Qin G, Zhang M, Chen N, Zhang M, Huang Y, Zhou J, Zhao Y, Liu M, Zhu X, Qiu Y, Sun Y, Huang C, Yan M, Wang M, Liu W, Tian F, Xu H, Zhou J, Wu Z, Shi T, Zhu W, Qin J, Xie L, Fan J, Qian X, He F, Chinese Human Proteome Project (CNHPP) Consortium (2019). Proteomics identifies new therapeutic targets of early-stage hepatocellular carcinoma. Nature.

[bib35] Kantarjian HM, O’Brien S, Cortes J (2013). Homoharringtonine/omacetaxine mepesuccinate: the long and winding road to food and drug administration approval. Clinical Lymphoma, Myeloma & Leukemia.

[bib36] Keenan AB, Jenkins SL, Jagodnik KM, Koplev S, He E, Torre D, Wang Z, Dohlman AB, Silverstein MC, Lachmann A, Kuleshov MV, Ma’ayan A, Stathias V, Terryn R, Cooper D, Forlin M, Koleti A, Vidovic D, Chung C, Schürer SC, Vasiliauskas J, Pilarczyk M, Shamsaei B, Fazel M, Ren Y, Niu W, Clark NA, White S, Mahi N, Zhang L, Kouril M, Reichard JF, Sivaganesan S, Medvedovic M, Meller J, Koch RJ, Birtwistle MR, Iyengar R, Sobie EA, Azeloglu EU, Kaye J, Osterloh J, Haston K, Kalra J, Finkbiener S, Li J, Milani P, Adam M, Escalante-Chong R, Sachs K, Lenail A, Ramamoorthy D, Fraenkel E, Daigle G, Hussain U, Coye A, Rothstein J, Sareen D, Ornelas L, Banuelos M, Mandefro B, Ho R, Svendsen CN, Lim RG, Stocksdale J, Casale MS, Thompson TG, Wu J, Thompson LM, Dardov V, Venkatraman V, Matlock A, Van Eyk JE, Jaffe JD, Papanastasiou M, Subramanian A, Golub TR, Erickson SD, Fallahi-Sichani M, Hafner M, Gray NS, Lin J-R, Mills CE, Muhlich JL, Niepel M, Shamu CE, Williams EH, Wrobel D, Sorger PK, Heiser LM, Gray JW, Korkola JE, Mills GB, LaBarge M, Feiler HS, Dane MA, Bucher E, Nederlof M, Sudar D, Gross S, Kilburn DF, Smith R, Devlin K, Margolis R, Derr L, Lee A, Pillai A (2018). The Library of Integrated Network-Based Cellular Signatures NIH Program: System-Level Cataloging of Human Cells Response to Perturbations. Cell Systems.

[bib37] Kim SM, Leem S-H, Chu I-S, Park Y-Y, Kim SC, Kim S-B, Park ES, Lim JY, Heo J, Kim YJ, Kim D-G, Kaseb A, Park YN, Wang XW, Thorgeirsson SS, Lee J-S (2012). Sixty-five gene-based risk score classifier predicts overall survival in hepatocellular carcinoma. Hepatology (Baltimore, Md.).

[bib38] Koleti A, Terryn R, Stathias V, Chung C, Cooper DJ, Turner JP, Vidovic D, Forlin M, Kelley TT, D’Urso A, Allen BK, Torre D, Jagodnik KM, Wang L, Jenkins SL, Mader C, Niu W, Fazel M, Mahi N, Pilarczyk M, Clark N, Shamsaei B, Meller J, Vasiliauskas J, Reichard J, Medvedovic M, Ma’ayan A, Pillai A, Schürer SC (2018). Data Portal for the Library of Integrated Network-based Cellular Signatures (LINCS) program: integrated access to diverse large-scale cellular perturbation response data. Nucleic Acids Research.

[bib39] Kong J, Lee H, Kim D, Han SK, Ha D, Shin K, Kim S (2020). Network-based machine learning in colorectal and bladder organoid models predicts anti-cancer drug efficacy in patients. Nature Communications.

[bib40] Kudo M, Finn RS, Qin S, Han K-H, Ikeda K, Piscaglia F, Baron A, Park J-W, Han G, Jassem J, Blanc JF, Vogel A, Komov D, Evans TRJ, Lopez C, Dutcus C, Guo M, Saito K, Kraljevic S, Tamai T, Ren M, Cheng A-L (2018). Lenvatinib versus sorafenib in first-line treatment of patients with unresectable hepatocellular carcinoma: a randomised phase 3 non-inferiority trial. Lancet (London, England).

[bib41] Lamb J, Crawford ED, Peck D, Modell JW, Blat IC, Wrobel MJ, Lerner J, Brunet J-P, Subramanian A, Ross KN, Reich M, Hieronymus H, Wei G, Armstrong SA, Haggarty SJ, Clemons PA, Wei R, Carr SA, Lander ES, Golub TR (2006). The Connectivity Map: using gene-expression signatures to connect small molecules, genes, and disease. Science (New York, N.Y.).

[bib42] Langfelder P, Horvath S (2008). WGCNA: an R package for weighted correlation network analysis. BMC Bioinformatics.

[bib43] Li B, Dewey CN (2011). RSEM: accurate transcript quantification from RNA-Seq data with or without a reference genome. BMC Bioinformatics.

[bib44] Li J, Zheng S, Chen B, Butte AJ, Swamidass SJ, Lu Z (2016). A survey of current trends in computational drug repositioning. Briefings in Bioinformatics.

[bib45] Liao Y, Smyth GK, Shi W (2014). featureCounts: an efficient general purpose program for assigning sequence reads to genomic features. Bioinformatics (Oxford, England).

[bib46] Liberzon A, Birger C, Thorvaldsdóttir H, Ghandi M, Mesirov JP, Tamayo P (2015). The Molecular Signatures Database (MSigDB) hallmark gene set collection. Cell Systems.

[bib47] Liu Z, Fang H, Reagan K, Xu X, Mendrick DL, Slikker W, Tong W (2013). In silico drug repositioning: what we need to know. Drug Discovery Today.

[bib48] Liu J, Lichtenberg T, Hoadley KA, Poisson LM, Lazar AJ, Cherniack AD, Kovatich AJ, Benz CC, Levine DA, Lee AV, Omberg L, Wolf DM, Shriver CD, Thorsson V, Hu H, Cancer Genome Atlas Research Network (2018). An Integrated TCGA Pan-Cancer Clinical Data Resource to Drive High-Quality Survival Outcome Analytics. Cell.

[bib49] Llovet JM, Zucman-Rossi J, Pikarsky E, Sangro B, Schwartz M, Sherman M, Gores G (2016). Hepatocellular carcinoma. Nature Reviews. Disease Primers.

[bib50] McCarthy DJ, Chen Y, Smyth GK (2012). Differential expression analysis of multifactor RNA-Seq experiments with respect to biological variation. Nucleic Acids Research.

[bib51] Mendez D, Gaulton A, Bento AP, Chambers J, De Veij M, Félix E, Magariños MP, Mosquera JF, Mutowo P, Nowotka M, Gordillo-Marañón M, Hunter F, Junco L, Mugumbate G, Rodriguez-Lopez M, Atkinson F, Bosc N, Radoux CJ, Segura-Cabrera A, Hersey A, Leach AR (2019). ChEMBL: towards direct deposition of bioassay data. Nucleic Acids Research.

[bib52] Meyers RM, Bryan JG, McFarland JM, Weir BA, Sizemore AE, Xu H, Dharia NV, Montgomery PG, Cowley GS, Pantel S, Goodale A, Lee Y, Ali LD, Jiang G, Lubonja R, Harrington WF, Strickland M, Wu T, Hawes DC, Zhivich VA, Wyatt MR, Kalani Z, Chang JJ, Okamoto M, Stegmaier K, Golub TR, Boehm JS, Vazquez F, Root DE, Hahn WC, Tsherniak A (2017). Computational correction of copy number effect improves specificity of CRISPR-Cas9 essentiality screens in cancer cells. Nature Genetics.

[bib53] Musa A, Ghoraie LS, Zhang S-D, Glazko G, Yli-Harja O, Dehmer M, Haibe-Kains B, Emmert-Streib F (2018). A review of connectivity map and computational approaches in pharmacogenomics. Briefings in Bioinformatics.

[bib54] Nakagawa S, Wei L, Song WM, Higashi T, Ghoshal S, Kim RS, Bian CB, Yamada S, Sun X, Venkatesh A, Goossens N, Bain G, Lauwers GY, Koh AP, El-Abtah M, Ahmad NB, Hoshida H, Erstad DJ, Gunasekaran G, Lee Y, Yu M-L, Chuang W-L, Dai C-Y, Kobayashi M, Kumada H, Beppu T, Baba H, Mahajan M, Nair VD, Lanuti M, Villanueva A, Sangiovanni A, Iavarone M, Colombo M, Llovet JM, Subramanian A, Tager AM, Friedman SL, Baumert TF, Schwarz ME, Chung RT, Tanabe KK, Zhang B, Fuchs BC, Hoshida Y, Precision Liver Cancer Prevention Consortium (2016). Molecular Liver Cancer Prevention in Cirrhosis by Organ Transcriptome Analysis and Lysophosphatidic Acid Pathway Inhibition. Cancer Cell.

[bib55] Niepel M, Hafner M, Duan Q, Wang Z, Paull EO, Chung M, Lu X, Stuart JM, Golub TR, Subramanian A, Ma’ayan A, Sorger PK (2017). Common and cell-type specific responses to anti-cancer drugs revealed by high throughput transcript profiling. Nature Communications.

[bib56] O’Rourke JM, Sagar VM, Shah T, Shetty S (2018). Carcinogenesis on the background of liver fibrosis: Implications for the management of hepatocellular cancer. World Journal of Gastroenterology.

[bib57] Parkkinen JA, Kaski S (2014). Probabilistic drug connectivity mapping. BMC Bioinformatics.

[bib58] Qiu Z, Li H, Zhang Z, Zhu Z, He S, Wang X, Wang P, Qin J, Zhuang L, Wang W, Xie F, Gu Y, Zou K, Li C, Li C, Wang C, Cen J, Chen X, Shu Y, Zhang Z, Sun L, Min L, Fu Y, Huang X, Lv H, Zhou H, Ji Y, Zhang Z, Meng Z, Shi X, Zhang H, Li Y, Hui L (2019). A Pharmacogenomic Landscape in Human Liver Cancers. Cancer Cell.

[bib59] Qu C, Zheng D, Li S, Liu Y, Lidofsky A, Holmes JA, Chen J, He L, Wei L, Liao Y, Yuan H, Jin Q, Lin Z, Hu Q, Jiang Y, Tu M, Chen X, Li W, Lin W, Fuchs BC, Chung RT, Hong J (2018). Tyrosine kinase SYK is a potential therapeutic target for liver fibrosis. Hepatology (Baltimore, Md.).

[bib60] Rees MG, Seashore-Ludlow B, Cheah JH, Adams DJ, Price EV, Gill S, Javaid S, Coletti ME, Jones VL, Bodycombe NE, Soule CK, Alexander B, Li A, Montgomery P, Kotz JD, Hon CS-Y, Munoz B, Liefeld T, Dančík V, Haber DA, Clish CB, Bittker JA, Palmer M, Wagner BK, Clemons PA, Shamji AF, Schreiber SL (2016). Correlating chemical sensitivity and basal gene expression reveals mechanism of action. Nature Chemical Biology.

[bib61] Ritchie ME, Phipson B, Wu D, Hu Y, Law CW, Shi W, Smyth GK (2015). limma powers differential expression analyses for RNA-sequencing and microarray studies. Nucleic Acids Research.

[bib62] Robin X, Turck N, Hainard A, Tiberti N, Lisacek F, Sanchez JC, Müller M (2011). pROC: an open-source package for R and S+ to analyze and compare ROC curves. BMC Bioinformatics.

[bib63] Roessler S, Long EL, Budhu A, Chen Y, Zhao X, Ji J, Walker R, Jia H-L, Ye Q-H, Qin L-X, Tang Z-Y, He P, Hunter KW, Thorgeirsson SS, Meltzer PS, Wang XW (2012). Integrative genomic identification of genes on 8p associated with hepatocellular carcinoma progression and patient survival. Gastroenterology.

[bib64] Scannell JW, Blanckley A, Boldon H, Warrington B (2012). Diagnosing the decline in pharmaceutical R&D efficiency. Nature Reviews. Drug Discovery.

[bib65] Scholten D, Trebicka J, Liedtke C, Weiskirchen R (2015). The carbon tetrachloride model in mice. Laboratory Animals.

[bib66] Schulze K, Imbeaud S, Letouzé E, Alexandrov LB, Calderaro J, Rebouissou S, Couchy G, Meiller C, Shinde J, Soysouvanh F, Calatayud A-L, Pinyol R, Pelletier L, Balabaud C, Laurent A, Blanc J-F, Mazzaferro V, Calvo F, Villanueva A, Nault J-C, Bioulac-Sage P, Stratton MR, Llovet JM, Zucman-Rossi J (2015). Exome sequencing of hepatocellular carcinomas identifies new mutational signatures and potential therapeutic targets. Nature Genetics.

[bib67] Setoain J, Franch M, Martínez M, Tabas-Madrid D, Sorzano COS, Bakker A, Gonzalez-Couto E, Elvira J, Pascual-Montano A (2015). NFFinder: an online bioinformatics tool for searching similar transcriptomics experiments in the context of drug repositioning. Nucleic Acids Research.

[bib68] Stathias V, Jermakowicz AM, Maloof ME, Forlin M, Walters W, Suter RK, Durante MA, Williams SL, Harbour JW, Volmar C-H, Lyons NJ, Wahlestedt C, Graham RM, Ivan ME, Komotar RJ, Sarkaria JN, Subramanian A, Golub TR, Schürer SC, Ayad NG (2018). Drug and disease signature integration identifies synergistic combinations in glioblastoma. Nature Communications.

[bib69] Subramanian A, Tamayo P, Mootha VK, Mukherjee S, Ebert BL, Gillette MA, Mesirov JP (2005). Gene set enrichment analysis: a knowledge-based approach for interpreting genome-wide expression profiles. PNAS.

[bib70] Subramanian A, Narayan R, Corsello SM, Peck DD, Natoli TE, Lu X, Gould J, Davis JF, Tubelli AA, Asiedu JK, Lahr DL, Hirschman JE, Liu Z, Donahue M, Julian B, Khan M, Wadden D, Smith IC, Lam D, Liberzon A, Toder C, Bagul M, Orzechowski M, Enache OM, Piccioni F, Johnson SA, Lyons NJ, Berger AH, Shamji AF, Brooks AN, Vrcic A, Flynn C, Rosains J, Takeda DY, Hu R, Davison D, Lamb J, Ardlie K, Hogstrom L, Greenside P, Gray NS, Clemons PA, Silver S, Wu X, Zhao W-N, Read-Button W, Wu X, Haggarty SJ, Ronco LV, Boehm JS, Schreiber SL, Doench JG, Bittker JA, Root DE, Wong B, Golub TR (2017). A Next Generation Connectivity Map: L1000 Platform and the First 1,000,000 Profiles. Cell.

[bib71] Tujebajeva RM, Graifer DM, Karpova GG, Ajtkhozhina NA (1989). Alkaloid homoharringtonine inhibits polypeptide chain elongation on human ribosomes on the step of peptide bond formation. FEBS Letters.

[bib72] Uhlén M, Fagerberg L, Hallström BM, Lindskog C, Oksvold P, Mardinoglu A, Sivertsson Å, Kampf C, Sjöstedt E, Asplund A, Olsson I, Edlund K, Lundberg E, Navani S, Szigyarto CA-K, Odeberg J, Djureinovic D, Takanen JO, Hober S, Alm T, Edqvist P-H, Berling H, Tegel H, Mulder J, Rockberg J, Nilsson P, Schwenk JM, Hamsten M, von Feilitzen K, Forsberg M, Persson L, Johansson F, Zwahlen M, von Heijne G, Nielsen J, Pontén F (2015). Proteomics. Tissue-based map of the human proteome. Science (New York, N.Y.).

[bib73] van Noort V, Schölch S, Iskar M, Zeller G, Ostertag K, Schweitzer C, Werner K, Weitz J, Koch M, Bork P (2014). Novel drug candidates for the treatment of metastatic colorectal cancer through global inverse gene-expression profiling. Cancer Research.

[bib74] Villa E, Critelli R, Lei B, Marzocchi G, Cammà C, Giannelli G, Pontisso P, Cabibbo G, Enea M, Colopi S, Caporali C, Pollicino T, Milosa F, Karampatou A, Todesca P, Bertolini E, Maccio L, Martinez-Chantar ML, Turola E, Del Buono M, De Maria N, Ballestri S, Schepis F, Loria P, Enrico Gerunda G, Losi L, Cillo U (2016). Neoangiogenesis-related genes are hallmarks of fast-growing hepatocellular carcinomas and worst survival. Results from a prospective study. Gut.

[bib75] Wagner A, Cohen N, Kelder T, Amit U, Liebman E, Steinberg DM, Radonjic M, Ruppin E (2015). Drugs that reverse disease transcriptomic signatures are more effective in a mouse model of dyslipidemia. Molecular Systems Biology.

[bib76] Wang M, Gong Q, Zhang J, Chen L, Zhang Z, Lu L, Yu D, Han Y, Zhang D, Chen P, Zhang X, Yuan Z, Huang J, Zhang X (2017). Characterization of gene expression profiles in HBV-related liver fibrosis patients and identification of ITGBL1 as a key regulator of fibrogenesis. Scientific Reports.

[bib77] Wang Z, Lachmann A, Keenan AB, Ma’ayan A (2018). L1000FWD: fireworks visualization of drug-induced transcriptomic signatures. Bioinformatics (Oxford, England).

[bib78] Wang H, Wang R, Huang D, Li S, Gao B, Kang Z, Tang B, Xie J, Yan F, Liang R, Li H, Yan J (2021). Homoharringtonine Exerts Anti-tumor Effects in Hepatocellular Carcinoma Through Activation of the Hippo Pathway. Frontiers in Pharmacology.

[bib79] Wei G, Twomey D, Lamb J, Schlis K, Agarwal J, Stam RW, Opferman JT, Sallan SE, den Boer ML, Pieters R, Golub TR, Armstrong SA (2006). Gene expression-based chemical genomics identifies rapamycin as a modulator of MCL1 and glucocorticoid resistance. Cancer Cell.

[bib80] Wen Q, O’Reilly P, Dunne PD, Lawler M, Van Schaeybroeck S, Salto-Tellez M, Hamilton P, Zhang S-D (2015). Connectivity mapping using a combined gene signature from multiple colorectal cancer datasets identified candidate drugs including existing chemotherapies. BMC Systems Biology.

[bib81] Wen Q, Kim CS, Hamilton PW, Zhang SD (2016). A gene-signature progression approach to identifying candidate small-molecule cancer therapeutics with connectivity mapping. BMC Bioinformatics.

[bib82] Weng T-Y, Wu HF, Li C-Y, Hung Y-H, Chang Y-W, Chen Y-L, Hsu H-P, Chen Y-H, Wang C-Y, Chang J-Y, Lai M-D (2018). Homoharringtonine induced immune alteration for an Efficient Anti-tumor Response in Mouse Models of Non-small Cell Lung Adenocarcinoma Expressing Kras Mutation. Scientific Reports.

[bib83] Wurmbach E, Chen Y, Khitrov G, Zhang W, Roayaie S, Schwartz M, Fiel I, Thung S, Mazzaferro V, Bruix J, Bottinger E, Friedman S, Waxman S, Llovet JM (2007). Genome-wide molecular profiles of HCV-induced dysplasia and hepatocellular carcinoma. Hepatology (Baltimore, Md.).

[bib84] Yang C, Chen J, Li Y, Huang X, Liu Z, Wang J, Jiang H, Qin W, Lv Y, Wang H, Wang C (2021). Exploring subclass-specific therapeutic agents for hepatocellular carcinoma by informatics-guided drug screen. Briefings in Bioinformatics.

[bib85] Yu G, Wang LG, Han Y, He QY (2012). clusterProfiler: an R package for comparing biological themes among gene clusters. OMICS.

[bib86] Zhang SD, Gant TW (2008). A simple and robust method for connecting small-molecule drugs using gene-expression signatures. BMC Bioinformatics.

[bib87] Zhao W, Li J, Chen M-JM, Luo Y, Ju Z, Nesser NK, Johnson-Camacho K, Boniface CT, Lawrence Y, Pande NT, Davies MA, Herlyn M, Muranen T, Zervantonakis IK, von Euw E, Schultz A, Kumar SV, Korkut A, Spellman PT, Akbani R, Slamon DJ, Gray JW, Brugge JS, Lu Y, Mills GB, Liang H (2020). Large-Scale Characterization of Drug Responses of Clinically Relevant Proteins in Cancer Cell Lines. Cancer Cell.

[bib88] Zhou WC, Zhang QB, Qiao L (2014). Pathogenesis of liver cirrhosis. World Journal of Gastroenterology.

[bib89] Zhou X, Wang M, Katsyv I, Irie H, Zhang B (2018). EMUDRA: Ensemble of Multiple Drug Repositioning Approaches to improve prediction accuracy. Bioinformatics (Oxford, England).

[bib90] Zhu AX, Kang Y-K, Yen C-J, Finn RS, Galle PR, Llovet JM, Assenat E, Brandi G, Pracht M, Lim HY, Rau K-M, Motomura K, Ohno I, Merle P, Daniele B, Shin DB, Gerken G, Borg C, Hiriart J-B, Okusaka T, Morimoto M, Hsu Y, Abada PB, Kudo M, REACH-2 study investigators (2019). Ramucirumab after sorafenib in patients with advanced hepatocellular carcinoma and increased α-fetoprotein concentrations (REACH-2): a randomised, double-blind, placebo-controlled, phase 3 trial. The Lancet. Oncology.

