# Supplementary material for: A survey of optimal strategy for signature-based drug repositioning and an application to liver cancer
Source: eLife. 2022 Feb 22;11:e71880. doi: 10.7554/eLife.71880 (PMC8893721; doi:10.7554/eLife.71880)

Raw unedited plots

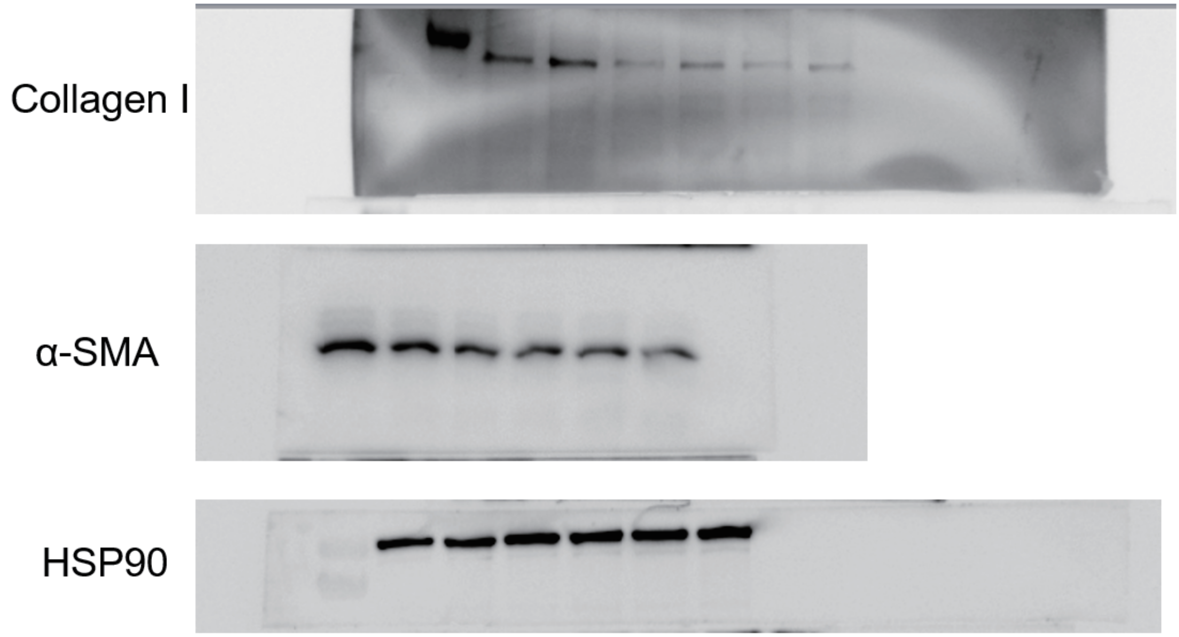

Labelled plots

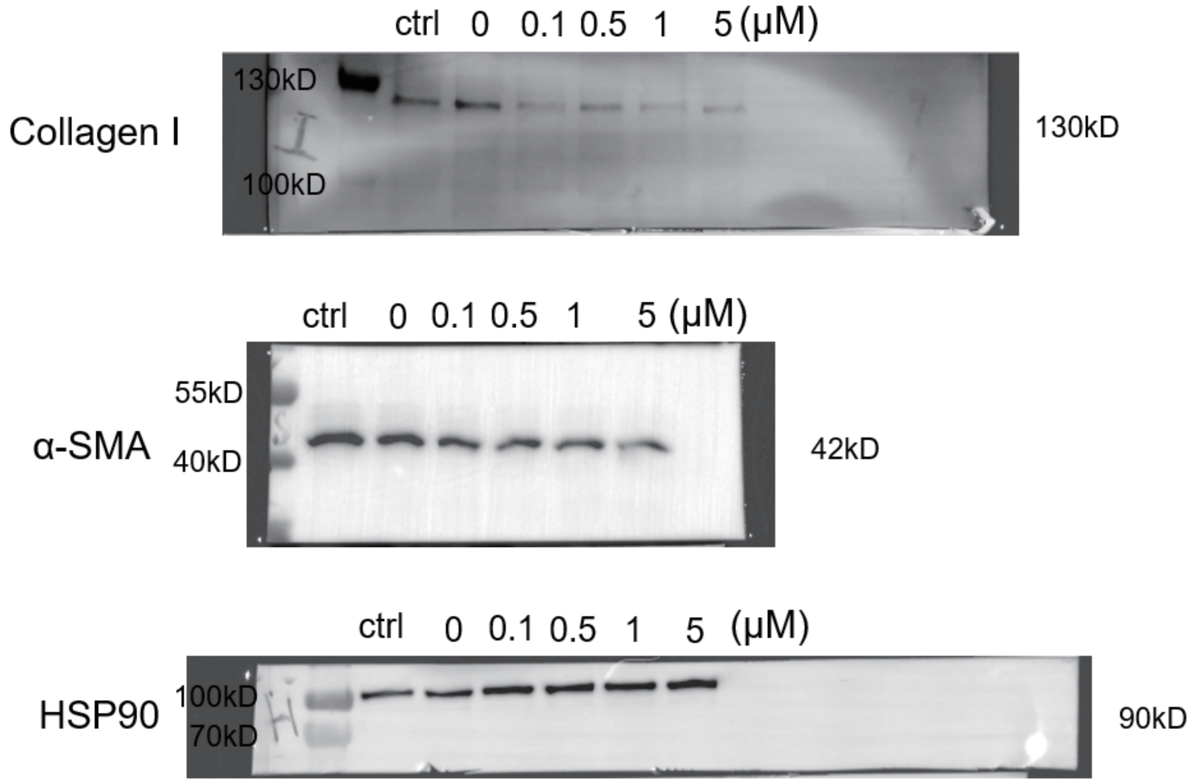

Supplement: Figure 8—source data 2. [file elife-71880-fig8-data2.pdf]
